# The *Saccharomyces cerevisiae* acetyltransferase Gcn5 exerts antagonistic pleiotropic effects on chronological ageing

**DOI:** 10.18632/aging.205109

**Published:** 2023-10-23

**Authors:** Kaiqiang Li, Gabriele Mocciaro, Jules L. Griffin, Nianshu Zhang

**Affiliations:** 1 Department of Biochemistry, University of Cambridge, Cambridge CB2 1GA, UK; 2 The Rowett Institute, University of Aberdeen, Foresterhill Campus, Aberdeen AB25 2ZD, UK

**Keywords:** Gcn5, SAGA, Hda1, chronological lifespan, antagonistic pleiotropy

## Abstract

Compared to replicative lifespan, epigenetic regulation of chronological lifespan (CLS) is less well understood in yeast. Here, by screening all the viable mutants of histone acetyltransferase (HAT) and histone deacetylase (HDAC), we demonstrate that Gcn5, functioning in the HAT module of the SAGA/SLIK complex, exhibits an epistatic relationship with the HDAC Hda1 to control the expression of starvation-induced stress response and respiratory cell growth. Surprisingly, the *gcn5Δ* mutants lose their colony-forming potential early in the stationary phase but display a longer maximum CLS than their WT counterparts, suggesting the contradictory roles of Gcn5 in lifespan regulation. Integrative analyses of the transcriptome, metabolome and ChIP assays reveal that Gcn5 is necessary for the activation of two regulons upon glucose starvation: the Msn2/4-/Gis1-dependent stress response and the Cat8-/Adr1-mediated metabolic reprogramming, to enable pro-longevity characteristics, including redox homeostasis, stress resistance and maximal storage of carbohydrates. The activation of Cat8-/Adr1-dependent regulon also promotes the pyruvate dehydrogenase (PDH) bypass, leading to acetyl-CoA synthesis, global and targeted H3K9 acetylation. Global H3K9 acetylation levels mediated by Gcn5 and Hda1 during the transition into stationary phase are positively correlated with senescent cell populations accumulated in the aged cell cultures. These data suggest that Gcn5 lies in the centre of a feed-forward loop between histone acetylation and starvation-induced gene expression, enabling stress resistance and homeostasis but also promoting chronological ageing concomitantly.

## INTRODUCTION

In *Saccharomyces cerevisiae*, two forms of ageing are modelled, including the replicative lifespan (RLS), which measures the maximum number of mitotic divisions a cell can undergo before senescence, and the chronological lifespan (CLS), which determines the period of time post-mitotic cells retain the ability to exit from quiescence [1, 2]. Apart from cytosine methylation which is absent in *S. cerevisiae* [3], other forms of chromatin remodelling have been found to regulate RLS in yeast [reviewed in 4, 5]. In contrast, epigenetic mechanisms linked to CLS regulation are poorly characterised, although some epigenetic regulatory enzymes associated with RLS also influence CLS. In this aspect, the removal of H3K36 demethylase Rph1 not only extends RLS [6], but also promotes CLS via a mitochondrion-to-nucleus signalling affecting gene repression at the subtelomeric regions [7]. Moreover, the evolutionarily conserved SAGA (Spt-Ada-Gcn5 acetyltransferase) complex has been associated with both CLS and RLS. The structural integrity of SAGA is required for both CLS and RLS extension, whereas the acetyltransferase activity encoded by *GCN5* only influences CLS but not RLS [8]. Intriguingly, *GCN5* has been shown to be essential for the retention of circular DNA within mother cells, causing the accumulation of nuclear pore complexes and disorganization of the nuclei of replicative ageing cells [9]. However, a number of studies have reported that deletion of *GCN5* compromises RLS extension mediated via the retrograde response [10, 11] or due to loss of deubiquitinase activity in SAGA [12]. More recently, Huang et al. [13] have revealed that inhibiting the histone acetyltransferase activity of Gcn5 or reducing *GCN5* expression by half in the heterozygous mutants significantly extends RLS. These studies suggest that Gcn5 may play contradictory roles in RLS extension. Similarly, removal of Gcn5 reduces CLS in synthetic medium but enhances CLS under winemaking conditions [14]. Thus, it remains unclear how Gcn5/SAGA regulate lifespan through its roles in histone acetylation and transcription regulation.

During the transition from fermentative growth (glucose-replete) to stationary phase (glucose-starved), yeast cells switch to respiratory growth on non-fermentable carbon sources, such as ethanol and glycerol, and acquire a set of characteristics typical of stationary-phase cells, including enhanced resistance to a variety of environmental stressors, the accumulation of storage carbohydrates (glycogen and trehalose), thickening of the cell wall and ultimately, the ability to maintain long-term survival [15–17]. We and others have previously reported that CLS extension in yeast is dependent on metabolic reprogramming to accumulate storage carbohydrates, especially trehalose, and the activation of the stress response program mediated in part by the stress response factors Msn2/Msn4 (Msn2/4) and the post-diauxic shift factor Gis1 [2, 18]. Metabolic reprogramming to store carbohydrates and the activation of the stress response program are coordinated by a number of signalling pathways. These include the Greatwall kinase Rim15 and the DYRK kinase Yak1 that are negatively regulated by the nutrient-sensitive TOR and PKA pathways [19, 20], the cell wall integrity pathway, the energy-sensing SNF1 complex and the Gsk-3 homologue Mck1 [21, 22]. To find the epigenetic factors that are involved in the metabolic reprogramming and/or the stress response program, we screened all the mutants of HAT (histone acetyltransferase) and HDAC (histone deacetylase) in the deletion library. As a result, we revealed that *GCN5*, functioning in the HAT module of the SAGA/SLIK complex, is epistatic to *HDA1*, encoding the catalytic subunit of the HDA1 complex to activate starvation-induced gene expression, respiratory cell growth, redox homeostasis and the accumulation of storage carbohydrates during the transition into stationary phase. Intriguingly, the *gcn5Δ* mutants lost their clonogenic potential early in the stationary phase but displayed a longer maximum lifespan than their WT counterparts, indicating the contradictory roles of Gcn5 in CLS regulation. Subsequent analyses of the transcriptome, metabolome and ChIP assays suggest that the readouts of the Gcn5-mediated histone acetylation not only promote the accumulation of pro-longevity characteristics and CLS extension, but also activate the PDH bypass, acetyl-CoA synthesis, and global histone acetylation, the latter of which is negatively associated with clonogenic survival among the aged cells. These findings revealed the multiple facets of histone acetylation in starvation-induced gene expression and their contrasting roles in CLS regulation, providing novel insights into the ageing mechanisms in non-dividing cells.

## RESULTS

### Gcn5 and Hda1 play opposing roles in starvation-induced HSP expression

**Figure 1:**
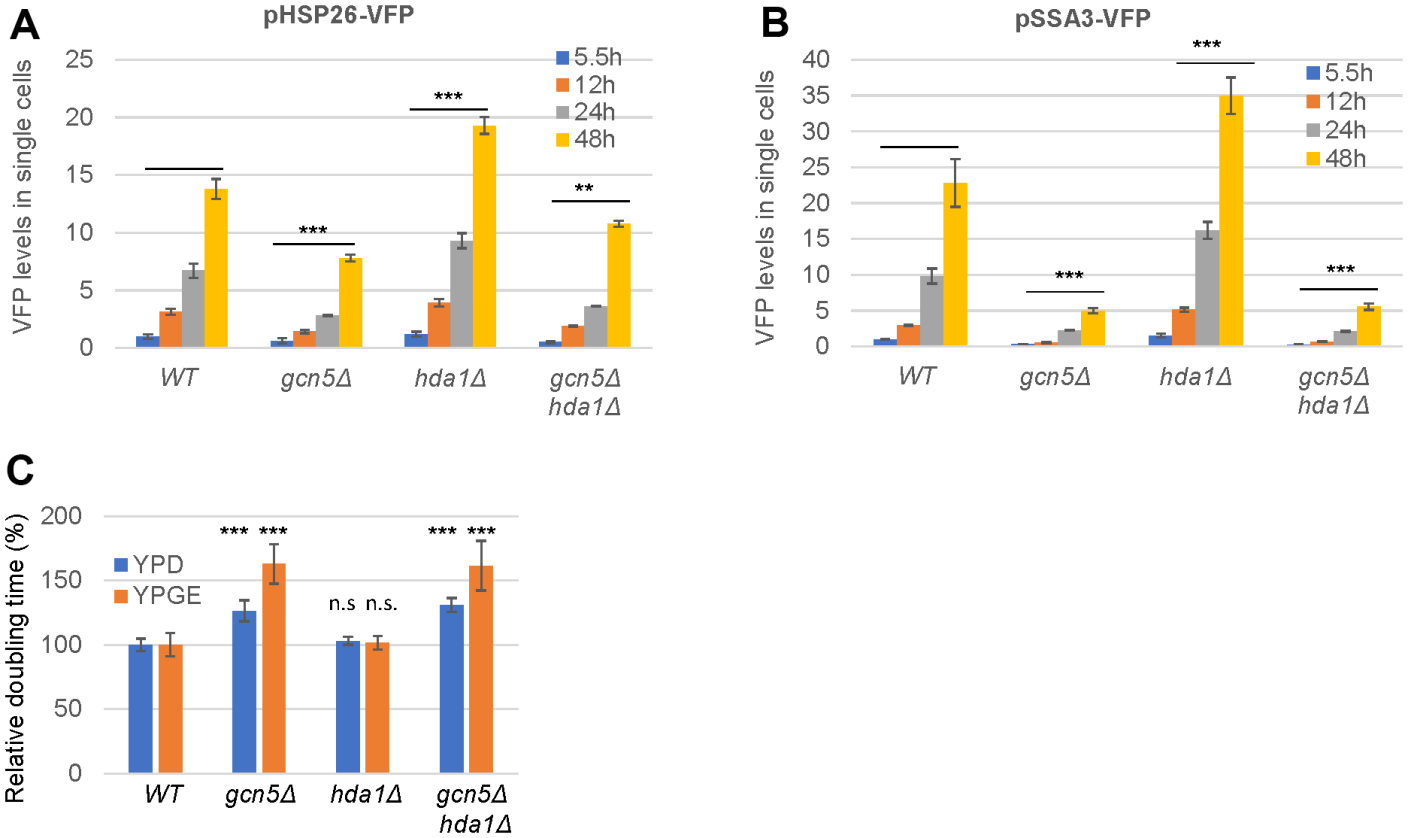
**Gcn5 exhibits an epistatic relationship with Hda1 in starvation-induced HSP expression and cell growth.** (**A**, **B**) Starvation-induced expression levels of pHSP26-VFP (**A**) and pSSA3-VFP (**B**); (**C**) Relative growth rates. YPD (2% glucose) and YPGE (3% glycerol and 1% ethanol). The levels of the HSP reporters in WT cells at exponential phase (5.5h) were set to 1. The significance of differences was revealed by two-factor ANOVA analysis for HSP reporters across all the timepoints (**A**, **B**) or by student’s t-test for growth rates (**C**). ***: p < 0.001, **: 0.001<p< 0.01, *: 0.01<p< 0.05 and n.s.: p> 0.05. Error bars represent standard deviation calculated from biological triplicates.

To find the epigenetic factors that are involved in starvation-induced stress response, we screened all the viable HDAC and HAT mutants from the BY4741 deletion library, using the heat shock protein (HSP) reporters, pHSP26-HSP26-RFP/VFP (a member of small HSP) and pSSA3-RFP/VFP (a member of the HSP70 family) [21]. Among the HDAC mutants, only the *hda1Δ*, *hda2Δ* and *hda3Δ* deletants displayed enhanced expression of both reporters (Supplementary Figure 1A, 1B), indicating that the HDA1 HDAC complex negatively influences the starvation-induced HSP expression. The HDA1 complex consists of the catalytic Hda1 homodimer, which interacts with the Hda2-Hda3 heterodimer to form an active tetramer to deacetylate histones H2B, H3 and H4. Disruption of any of the three subunits has been shown to similarly abolish the catalytic activity of the HDA1 complex both *in vitro* and *in vivo* [23]. Hence, we only include the *hda1Δ* mutant for further investigation. Among the HAT mutants, removal of *GCN5*, encoding the catalytic subunit of the SAGA/ADA/SLIK complexes, led to significantly reduced levels of both reporters (Supplementary Figure 1C, 1D). Intending to ensure the same genetic background, the *gcn5Δ* and *hda1Δ* deletion mutants were regenerated from BY4742 cells. As shown in Figure 1A, 1B, starvation-induced HSP expression was dramatically reduced in the *gcn5Δ* mutants and significantly enhanced in the *hda1Δ* cells. Further removal of *HDA1* has no impact on the levels of either reporter observed in the *gcn5Δ* mutants (Figure 1A, 1B), suggesting an epistatic relationship between them. Moreover, in comparison to pHSP26-VFP, starvation-induced pSSA3-VFP is expressed to a relatively higher level in WT or *hda1∆* cells and is more severely reduced in the *gcn5∆* or *gcn5∆hda1∆* mutants (note the different scales in Y axis of Figure 1A, 1B), suggesting that the acetylation status has a more significant influence on the expression of *SSA3* than that of *HSP26*. The *gcn5Δ* mutants were previously shown to have respiratory growth defects [24]. The moderate fermentative or more severe respiratory growth defects exhibited by the *gcn5Δ* cells were not rescued by *HDA1* removal (Figure 1C). These data imply that the roles of the HDA1 complex in the regulation of starvation-induced gene expression are dependent on Gcn5.

### Gcn5 functions in the HAT module of SAGA to promote HSP expression and respiratory growth

**Figure 2:**
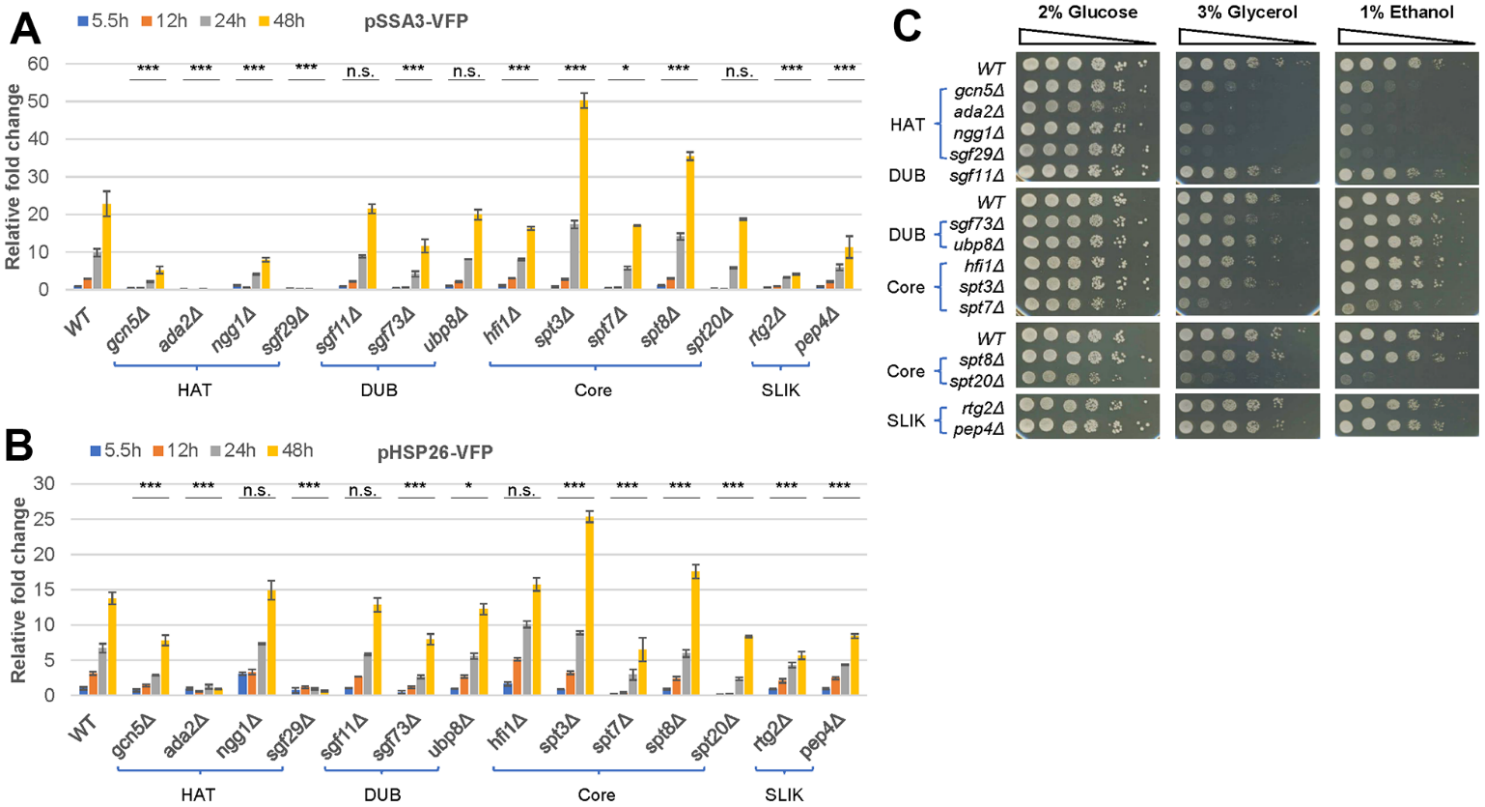
**Gcn5 functions within the HAT module of the SAGA/SLIK complex to promote starvation-induced gene expression and respiratory growth.** (**A**, **B**) Relative expression levels of pSSA3-VFP (**A**) and pHSP26-VFP (**B**) in mutants of the SAGA/SLIK complexes; (**C**) Spotting assays of cell growth on YPD (2% glucose), YPG (3% glycerol) and YPE (1% Ethanol) media. HAT, histone acetyltransferase module; DUB, deubiquitinase module; Core, Core module; SLIK, SAGA-like complex. Expression levels of the two reporters in WT cells at 5.5h post-inoculation was set to 1. Error bars represent standard deviation calculated from biological triplicates. The significance of difference between *WT* and mutants across all time points was revealed by two-factor ANOVA analysis. ***: p < 0.001, **: 0.001<p< 0.01, *: 0.01<p< 0.05 and n.s.: p> 0.05.

Gcn5 only acetylates nucleosomal histones efficiently when functioning within multi-subunit complexes. To find which complex (Supplementary Figure 2A) is responsible for the stress response and/or respiratory growth, we next screened all the viable mutants of the three complexes in the BY4742 library. Deletion of any of the HAT module subunits (*GCN5*, *ADA2*, *NGG1*, and *SGF29*) led to dramatic reduction of pSSA3-VFP (Figure 2A) and pHSP26-VFP (Figure 2B, except for *ngg1Δ*) and severe respiratory growth defects (Figure 2C), supporting that the integrity of the HAT module is essential to the activation of stress response and respiratory growth. However, removal of the ADA-specific subunits resulted in moderately reduced pSSA3-VFP expression only in *ahc2Δ* (Supplementary Figure 2B) but little impact on pHSP26-VFP expression (Supplementary Figure 2C) or respiratory growth (Supplementary Figure 2D). These data suggest that the ADA complex may play only a minor role in starvation-induced gene expression. In contrast, deletion of the SAGA-specific subunit *SPT7* significantly reduced the expression levels of both reporters (Figure 2A, 2B) and respiratory growth on either glycerol or ethanol (Figure 2C), similar to those observed for *gcn5Δ*. Moreover, removal of SAGA/SLIK-specific *SPT3* or the SAGA-specific *SPT8* significantly enhanced the expression levels of the two reporters (Figure 2A, 2B), consistent with their roles in preventing SAGA from interaction with the transcriptional machinery [25, 26]. The SLIK complex contains a unique subunit Rtg2 [27] and a truncated version of the Spt7 modified by the proteinase Pep4 [28]. Removal of SLIK-specific *RTG2* or *PEP4* resulted in significant reduction of both reporters (Figure 2A, 2B). However, unlike *gcn5Δ* mutants, *rtg2Δ* and *pep4Δ* mutants displayed no observable respiratory growth defects (Figure 2C). Put together, these data suggest that Gcn5 is likely to function within the HAT module of the SAGA/SLIK complex to regulate the Msn2/4- and Gis1-mediated stress response. Meanwhile, Gcn5 may function in the HAT module of SAGA to promote respiratory growth.

Within the HAT module of SAGA/SLIK complexes, Gcn5 acts as the catalytic subunit. Ada2 functions to promote the HAT activity of Gcn5, while Ngg1 plays a role in expanding the range of lysine residues that undergo acetylation to allow robust nucleosomal histone acetylation [29]. Deletion of *NGG1* led to similarly reduced pSSA3-VFP as observed in the *gcn5∆* mutants (Figure 2A) but had no impact on the expression of pHSP26-VFP (Figure 2B), suggesting that nucleosomal histone acetylation is necessary for the expression of *SSA3* and may not be required for *HSP26* induction. Furthermore, Ada2 is essential for the proper binding of Gcn5-containing complexes to histone H3 N-terminal tail [30]. The exact roles of Sgf29 remained unclear, although preliminary data indicated that Sgf29 can recognise and bind to H3K4me3, hence recruiting Gcn5-containing complexes to the N-terminus of histone H3 to stimulate histone acetylation [29]. Therefore, while deletion of *GCN5* and *NGG1* abolishes and significantly impairs the HAT activity respectively, loss of either *ADA2* or *SGF29* may completely prevent the binding of Gcn5-containing complexes to the N-terminus of histone H3. The more severely reduced expression of both reporters observed in *ada2Δ* and *sgf29Δ* (Figure 2A, 2B) cells may suggest that the SAGA/SLIK complex (even without the HAT activity) is necessary for optimal gene expression upon glucose starvation.

### Gcn5 displays contradictory roles in the regulation of CLS

**Figure 3:**
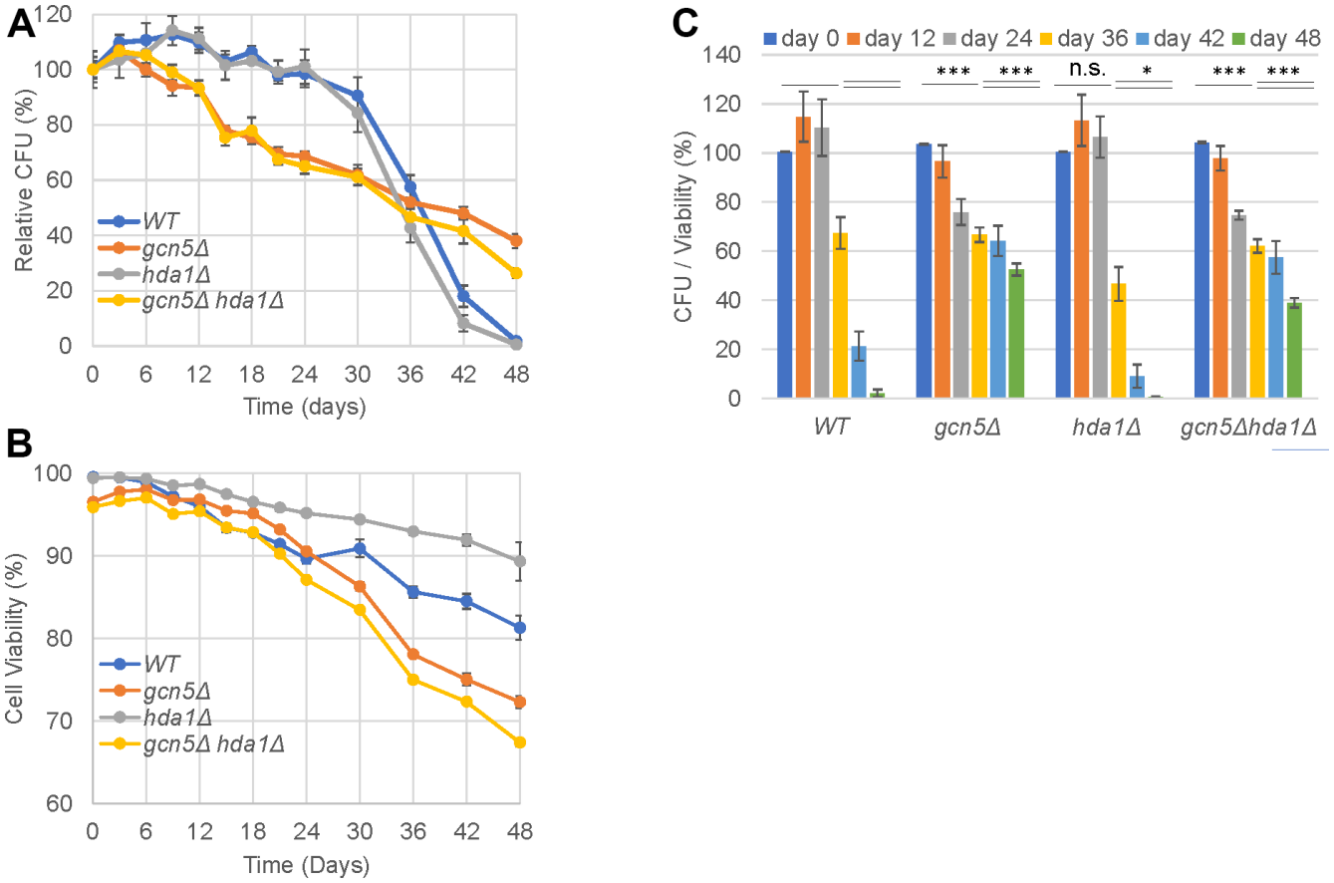
**Gcn5 displays contradictory roles in the regulation of chronological lifespan.** (**A**) Relative CFU; (**B**) Cell viability; (**C**) Normalised CFU to cell viability. Two-factor ANOVA analysis was conducted to reveal the significance of differences across the indicated timepoints. ***: p < 0.001, **: 0.001<p< 0.01, *: 0.01<p< 0.05 and n.s.: p> 0.05.

Next, we investigated the roles of Gcn5 in chronological lifespan extension by measuring the ability of stationary-phase cells to exit quiescence (colony-forming units, CFU). Simultaneously, cell viability was measured by nuclear DNA staining using Sytox Green [21] in cells that have lost their membrane integrity [31]. Comparing CFU with cell viability indicates the relative levels of viable cells capable of quiescence exit, or oppositely, the levels of cellular senescence. Compared to WT, the *gcn5Δ* mutants lost their colony-forming ability much faster in the early- to mid-stationary phase (≤24 days, Figure 3A). However, such CFU loss in *gcn5Δ* mutants was constant and more slowly than that seen in WT cells towards the late stationary phase (≥30days, Figure 3A) when the latter started to lose their CFU rapidly. In contrast, the *gcn5Δ* mutants had similar cell viability (>85%) as WT cells during the early- to mid-stationary phase (≤24 days, Figure 3B) and lost their viability faster than WT cells thereafter (≥30 days, Figure 3B). However, the absolute difference of cell viability between WT cells and the *gcn5Δ* mutants is within 10% until the end of the observational period (note that the lower limit on the Y axis is 60% in Figure 3B). Relative CFU to cell viability for the *gcn5Δ* mutants was reduced from 100% to ~70% in the first 24 days and at a similar rate from ~70% to ~50% between 24 and 48 days (Figure 3C). In contrast, relative CFU potential for WT cells was decreased from 100% to nearly 0 during the latter period (Figure 3C). These data indicate that Gcn5 plays contradictory roles in the regulation of CFU potential of the stationary phase cells. Removal of *GCN5* results in early but slower rate of chronological ageing, thus a longer maximum lifespan than that of WT cells.

Compared to WT cells, the *hda1Δ* mutants did not display an improved ability to form colonies (Figure 3A), as opposed to that demonstrated by Yu et al. [32]. One possibility to account for the differences could be the dramatically different CFU of WT cells used in the two studies. Relative CFU of the WT cells in our study continued to increase until day 9 and did not decrease below 100% until day 21 (Figure 3A), whereas the relative CFU of WT cells in the study by Yu et al. did not increase during early stationary phase and started to decrease after day 10. Thus, the likely CFU improvement as a result of Hda1 removal [32] may not be revealed in our study. Nevertheless, Hda1 removal did lead to increased cell viability across the entire stationary phase tested (Figure 3B). Normalising CFU to cell viability indicated that Hda1 removal resulted in reduced ability of viable cells to exit quiescence towards the late-stationary phase (≥30 days, Figure 3C), indicating enhanced senescent cell population in the aged cultures. Further removal of *HDA1* did not significantly influence the clonogenic survival (Figure 3A), cell viability (Figure 3B) or the CFU/viability ratio (Figure 3C) of the *gcn5Δ* mutants, further suggesting the epistatic relationship between *GCN5* and *HDA1* in CLS regulation.

### Gcn5 transcriptionally promotes metabolic reprogramming and inhibits ribosome biogenesis upon glucose starvation

**Figure 4:**
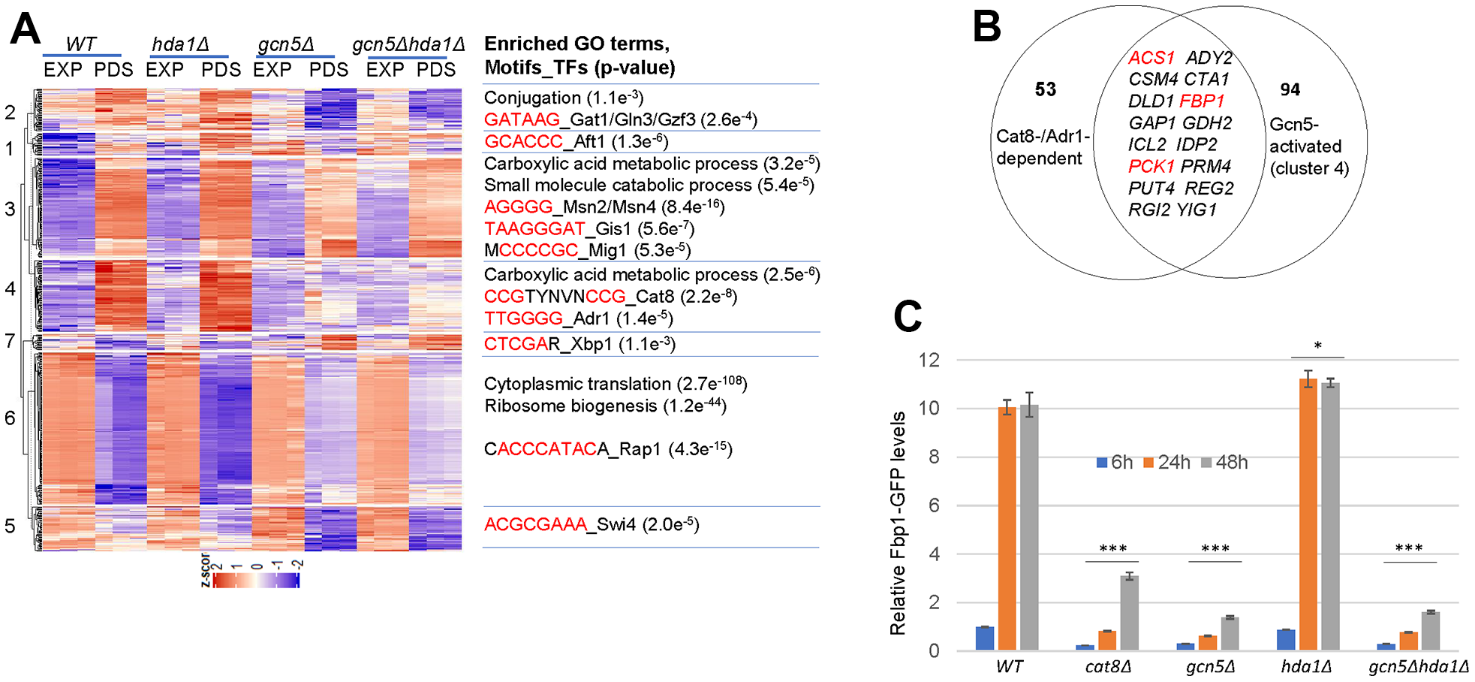
**Gcn5 transcriptionally promotes starvation-induced stress response and respiratory metabolisms.** (**A**) Hierarchical clustering of differentially expressed genes regulated by *GCN5* at PDS phase (n=594, net log2 Fold change >= 1). (**B**) A pie chart showing the overlap genes between Cat8-/Adr1-targets and those dependent on Gcn5 in cluster 4. (**C**) Relative Fbp1-GFP levels in cells grown to glucose starvation; The significance of differences was revealed by two-factor ANOVA analysis for Fbp1-GFP reporters across all the timepoints. ***: p < 0.001, **: 0.001<p< 0.01, *: 0.01<p< 0.05 and n.s.: p> 0.05. Error bars represent standard deviation calculated from biological triplicates.

Intending to find how Gcn5 may regulate CLS extension contradictorily, we first conducted RNA-seq analyses of the transcriptome isolated from exponentially-growing (EXP, glucose-replete) and early post-diauxic shift (PDS, glucose-depleted) cells. Differential expression (DE) analysis identified 594 genes whose transcription is significantly regulated (>2-fold) in glucose-depleted *gcn5Δ* cells. Surprisingly, ~42% of these DE genes were upregulated and the rest downregulated in PDS *gcn5Δ* cells (Figure 4A), suggesting that Gcn5 is implicated in transcription activation (clusters 1, 2, 3, 4 and 5, Figure 4A and Supplementary Table 1) as well as transcriptional repression (clusters 6 and 7, Figure 4A and Supplementary Table 1). Apart from cluster 1, the *hda1Δ* mutants displayed similar gene expression patterns as WT cells in all other clusters and the expression of these DE genes were similarly regulated in the *gcn5Δ* and *gcn5Δhda1Δ* mutants (Figure 4A), supporting that the roles of *HDA1* in glucose starvation-induced gene expression are largely dependent on *GCN5*.

Enriched GO terms, motifs in the promoter regions [33] and potential transcription factors (TFs) regulating their expression [34] were identified for each gene cluster (Figure 4A). GO analysis indicated that ~50% and ~40% of the 201 genes in cluster 6 were involved in cytoplasmic translation and ribosome biogenesis (RiBi) respectively (Figure 4A), indicating that *GCN5* is necessary for global repression of translation in response to glucose depletion. Promoter analysis revealed that the most significantly overrepresented motif is the consensus sequence targeted by Rap1, the transcription factor required for expression of ribosomal protein genes (RPGs) and RiBi genes [35]. Maximal expression of RPGs also requires RPG-specific transcription activators Fhl1 and Ifh1, the latter of which has been shown to be rapidly released from target promoters under the stress conditions or TORC1 inhibition to downregulate RPG expression [35]. In this regard, Gcn5-mediated acetylation of Ifh1 negatively modulates the transcriptional activity of Ifh1 [36, 37]. Thus, removal of Gcn5 would lead to insufficient inhibition of Ifh1 and hence moderate expression of RPG and RiBi genes in glucose-depleted cells (cluster 6). Furthermore, a number of genes implicated in the glycolysis pathway, including *HXT2*, *HXT3*, *HXK2*, *PFK27*, *TDH2*, *GPM1*, *ENO2* and *CDC19*, were also found in cluster 6. Intriguingly, the transcription of these glycolytic genes also requires Rap1 [38, 39]. These data suggest that Gcn5 may target transcription factors directly to repress Rap1-mediated gene expression in response to glucose depletion.

Similar bioinformatic analyses indicated that Gcn5 promotes starvation-induced stress response and carboxylic acid metabolic process (clusters 3 and 4 in Figure 4A). Among the top 20 genes of cluster 3 (Table 1), many encode chaperones or hydrophilins (*GRE1*, *HSP32*, *HSP33*, *SIP18*, *SNO4* and *SPG1*) that are necessary for survival under stress conditions and a few are implicated in peroxisomal and mitochondrial metabolisms (*CYB2, POX1*, *FOX2* and *SHH4*). We and others have previously demonstrated that starvation- or rapamycin treatment-induced expression of heat shock proteins and hydrophilins, such as *GRE1* and those reporter genes (*SSA3* and *HSP26*) used to identify *GCN5* and *HDA1* in Figure 1 were mediated by the stress response transcription factors Msn2/4 and the post-diauxic shift factor Gis1 [40–45]. Indeed, consensus sequences targeted by Msn2/4 and Gis1 were significantly enriched in the promoter regions of cluster 3 genes (Figure 4A), supporting that Gcn5 is necessary to activate starvation-induced stress response mediated by both Msn2/4 and Gis1. Similarly, motifs targeted by the carbon source-responsive (CSRE) transcription factors Adr1 and Cat8 were overrepresented in the promoter sequences of cluster 4 genes (Figure 4A). Among the group of 53 genes that were activated by Cat8 and/or Adr1 [46], 16 were found in cluster 4 (Figure 4B), including those involved in gluconeogenesis (*FBP1* and *PCK1*) and acetyl-CoA synthesis (*ACS1*). Cluster 4 genes also include those implicated in the TCA cycle (*ACO1*, *IDP1*, *KGD1*, *LSC2*, *FUM1*), the PDH bypass (*ADH2*, *ALD6* and *ACS1*) (Supplementary Figure 3) and in oxidative phosphorylation (Supplementary Figure 4).

**Table 1:** Top 20 Gcn5-regulated genes in cluster 3 of Figure 4A.

**Gene name**	**Description**
*CYB2*	Cytochrome b2
*GRE1*	Hydrophilin
*SNO4*	Chaperone and cysteine protease
*DPC7*	Unknown function
*ATG39*	Autophagy receptor
*HSP32*	Chaperone and cysteine protease
*SPG1*	Required for high temperature survival during stationary phase
*SIP18*	Phospholipid-binding hydrophilin
*PAU22*	Unknown function
*YLR157W-D*	Unknown function
*SHC1*	Chitin synthase III activator
*YLR161W*	Unknown function
*PAU11*	Unknown function
*YGL260W*	Unknown function
*PAI3*	Pep4p inhibitor
*POX1*	Fatty-acyl coenzyme A oxidase
*FOX2*	3-hydroxyacyl-CoA dehydrogenase and enoyl-CoA hydratase
*SHH4*	Putative alternate subunit of succinate dehydrogenase
*SPS100*	Protein required for spore wall maturation
*HSP33*	Chaperone and cysteine protease

To further confirm that Gcn5 is involved in Cat8/Adr1-dependent gene expression, Fbp1-GFP fusion protein under the control of the endogenous *FBP1* promoter was expressed in WT and mutant cells grown to glucose starvation. As demonstrated previously [46], derepression of Fbp1-GFP in response to glucose depletion was dependent on *CAT8* (comparing 24h/48h to 6h, Figure 4C). Compared to WT cells, the induced levels of Fbp1-GFP were slightly higher in the *hda1Δ* mutants but nearly abolished in the *gcn5Δ* single or *gcn5Δhda1Δ* double mutants (Figure 4D), confirming that Gcn5 is essential to the activation of Cat8/Adr1-dependent gene expression in response to glucose starvation, whereas Hda1 has only a marginal impact. Transcription activation of Cat8-dependent genes is mediated by the energy-sensing Snf1 complex in glucose-depleted cells [47, 48]. Furthermore, H3 phosphorylation by Snf1 has been shown to precede SAGA recruitment and chromatin remodelling to activate Cat8- and Adr1-dependent transcription [49–51]. Put together, these and our data suggest that in response to glucose depletion, Gcn5 may function to repress the expression of the translational machinery and as the catalytic subunit of the SAGA/SLIK complexes, to remodel chromatin to activate a number of regulons: the stress response program mediated by Msn2/4 and Gis1, and the respiratory metabolisms promoted by the Snf1 complex and its downstream activators Cat8 and Adr1.

### Gcn5 enables redox homeostasis and stress resistance to promote CLS extension

**Figure 5:**
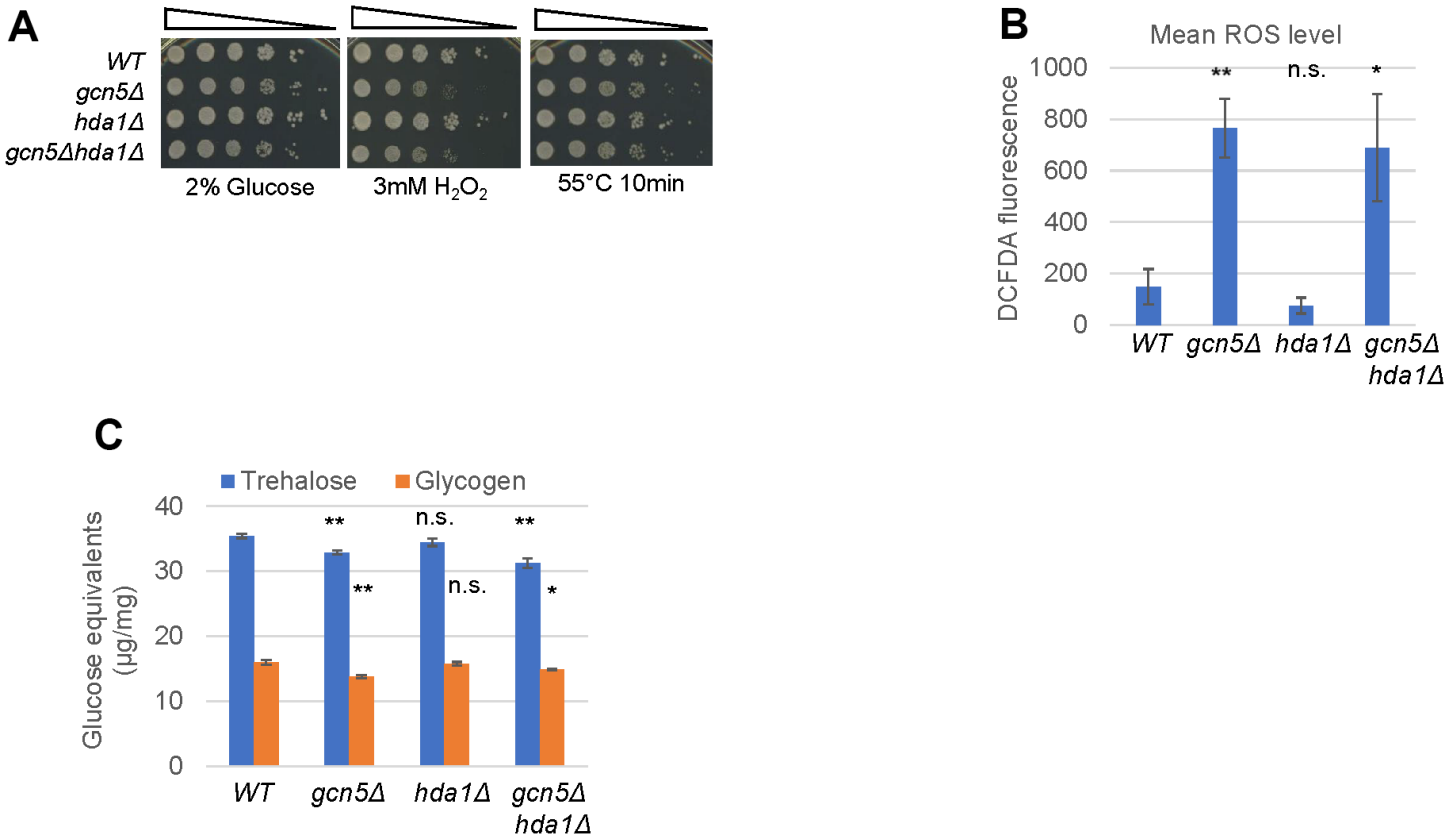
**Gcn5 is necessary for redox homeostasis and stress resistance.** (**A**) Oxidative and heat shock stress resistance displayed by early-stationary phase cells, (**B**) Mean ROS levels and (**C**) Trehalose and Glycogen accumulated in early-stationary phase cells. Error bar represents standard deviation calculated from biological triplicates. The significance of difference between *WT* and mutants was calculated by student’s t-test. ***: p < 0.001, **: 0.001<p< 0.01, *: 0.01<p< 0.05 and n.s.: p> 0.05.

Next, we assayed the phenotypes of the *gcn5Δ* mutants which may be associated with CLS extension. Early-stationary phase *gcn5Δ* cells displayed severe defects in resisting exogenous oxidative stress (Figure 5A), corresponding to their significantly increased intracellular ROS levels (Figure 5B). In comparison, ROS accumulation was insignificantly reduced in the *hda1Δ* mutant (Figure 5B). Further removal of *HDA1* did not influence the levels of intracellular ROS (Figure 5B) or the defects to resist oxidative stress in *gcn5Δ* cells (Figure 5A). In agreement with enhanced intracellular ROS accumulation, the transcript levels of mitochondrial superoxide dismutase *SOD2* and catalase *CTA1* were significantly impaired in starved cells bearing *gcn5Δ* (Supplementary Figure 5), suggesting that the anti-oxidant defence system is impaired in the *gcn5Δ* mutants. Similarly, early-stationary phase *gcn5Δ* and *gcn5Δhda1Δ* mutants display minor defects in heat resistance (Figure 5A) and marginally reduced trehalose and glycogen accumulation (Figure 5C). These data suggest that Gcn5 is necessary to promote CLS extension through its roles in the expression of molecular chaperones (Figure 1 and Table 1), the anti-oxidant defence system and optimal accumulation of trehalose to maintain redox homeostasis and resistance to stress conditions.

### Gcn5 is required for acetyl-CoA synthesis and histone acetylation on target gene promoters

**Figure 6:**
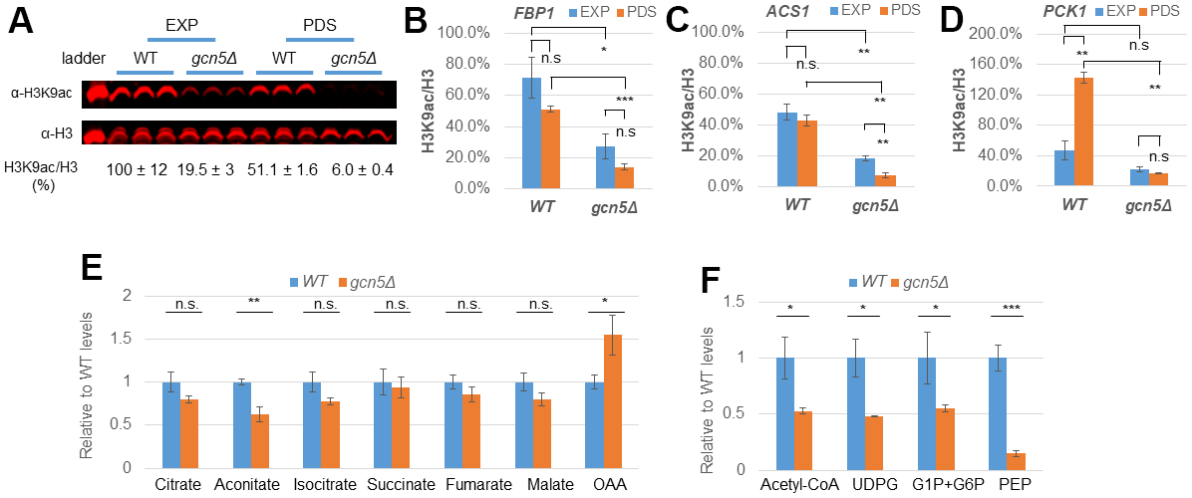
**Gcn5 is necessary for UDPG and Acetyl-CoA synthesis and H3K9 acetylation in glucose-depleted cells.** (**A**) Global H3K9 acetylation levels in *WT* and *gcn5Δ* cells; (**B**–**D**) H3K9 acetylation levels in promoters of *FBP1* (**B**), *ACS1* (**C**) and *PCK1* (**D**). (**E**, **F**) Relative levels of the TCA cycle metabolites (**E**) and Acetyl-CoA, UDPG, G1P and G6P together, and PEP (**F**) measured using LC-MS/MS. Student’s t-test was performed to reveal the differences between *WT* and *gcn5Δ* mutants or between EXP and PDS phases. ***: p < 0.001, **: 0.001<p< 0.01, *: 0.01<p< 0.05 and n.s.: p> 0.05. Error bars represent standard deviation calculated from biological triplicates. Abbreviation: EXP: exponential phase, PDS: early post-diauxic shift phase; OAA: oxaloacetate; UDPG: UDP-Glucose; G1P: Glucose-1-phosphate; G6P: Glucose-6-phosphate; PEP: Phosphoenolpyruvate.

To confirm that Gcn5/SAGA may be recruited to regulate respiratory metabolisms, we next interrogated the levels of histone acetylation at the target gene promoters through chromatin immunoprecipitation (ChIP) analysis. Histone H3 lysine 9 (H3K9) is a well-known target of Gcn5 within the SAGA complex [52, 53]. Overall H3K9ac levels were decreased by two-fold in WT cells transitioned from glucose-replete (EXP) to glucose-depleted conditions (PDS, Figure 6A). In comparison, H3K9ac levels between the two conditions remained similar at the promoter regions of *FBP1* (Figure 6B) and *ACS1* (Figure 6C), or significantly increased at the *PCK1* promoter (Figure 6D) in PDS cells. Strikingly, H3K9ac levels at any of the promoter regions were significantly reduced in the *gcn5Δ* deletants at the glucose-replete condition and more severely decreased upon glucose depletion (Figure 6B–6D). Recently, Gcn5/SAGA was shown to interact with Cat8 and Adr1 transcription factors to activate gluconeogenic and fat metabolism genes in cells subject to acute glucose starvation [54]. Our data suggest that Gcn5/SAGA is not only necessary for the basal levels of genome-wide H3K9 acetylation but also recruited to maintain or to enhance H3K9ac levels at the target genes in response to glucose depletion and when cells were grown on non-fermentable carbon sources. Furthermore, in contrast to fat metabolisms activated under acute glucose withdrawal [54], the PDH bypass pathway may be important for the provision of Acetyl-CoA in cells transitioned from fermentative growth to stationary phase (see next paragraph).

We next assessed the metabolome extracted from glucose-depleted cells using LC-MS/MS. In comparison to WT, the levels of the TCA cycle intermediates apart from aconitate and oxaloacetic acid (OAA) were not significantly changed (Figure 6E), suggesting that the flux through the TCA cycle was largely maintained despite transcription downregulation of most of the TCA cycle genes (Supplementary Figure 3). The significant increase of OAA (Figure 6E) and the corresponding decrease of phosphoenolpyruvate (PEP), glucose-6P and glucose-1P together, and UDPG (Figure 6F) support that Gcn5 is necessary for the activation of gluconeogenesis (Figure 4 and Supplementary Figure 3). Furthermore, the levels of acetyl-CoA were severely reduced in the *gcn5Δ* mutants depleted for glucose (Figure 6F), in agreement with transcription downregulation of the cytosolic PDH bypass leading to acetyl-CoA synthesis (Supplementary Figure 3). These findings lend further support to the conclusion from the transcriptome analysis that Gcn5 is required for the Cat8-/Adr1-dependnet gluconeogenesis and PDH bypass pathways to enable metabolic reprogramming in response to glucose starvation. *ACS1* encodes the aerobic isoform of acetyl-CoA synthase necessary to maintain nucleo-cytosolic acetyl-CoA levels and respiratory growth [55, 56]. These and our data also imply that Gcn5 may lie at the centre of a feed-forward loop to promote acetyl-CoA synthesis, histone acetylation, the stress response program and respiratory metabolisms.

### Global H3K9 acetylation mediated by Gcn5 and Hda1 is correlated with chronological senescence

**Figure 7:**
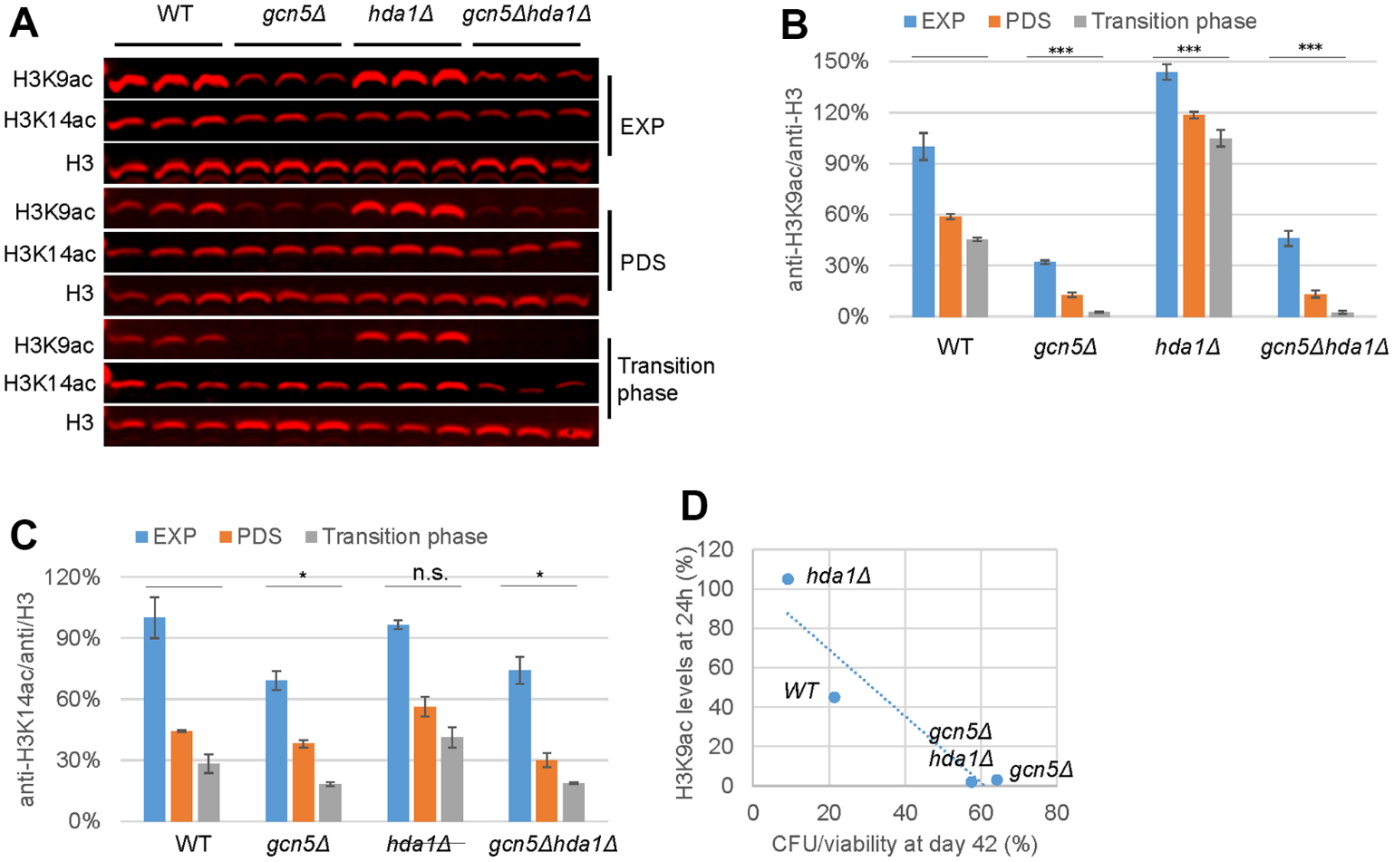
**H3K9 acetylation levels mediated by Gcn5 and Hda1 are correlated with senescent populations accumulated in the ageing cell cultures.** (**A**) Global H3K9ac and H3K14ac levels in *WT* and mutant cells; (**B**, **C**) Quantification of H3K9ac (**B**) and H3K14ac (**C**); (**D**) Correlation between H3K9ac levels with CFU/viability ratios. Two-factor ANOVA was performed to reveal the differences between *WT* and the mutants across different growth phases. ***: p < 0.001, **: 0.001<p< 0.01, *: 0.01<p< 0.05 and n.s.: p> 0.05. Error bars represent standard deviation calculated from biological triplicates. Abbreviation: EXP: exponential phase (6h), PDS: early post-diauxic shift phase (14h) and the transition phase (24h).

To determine the connection between Gcn5-mediated histone acetylation and its negative roles in CLS regulation (Figure 3A), we next assessed the global H3K9ac and H3K14ac levels in cells transitioned into the stationary phase. The levels of H3K9ac, which were previously shown to decrease by ~50% in *WT* cells in response to glucose depletion (Figure 5A), were further but more modestly reduced during the transition into the early-stationary phase (Figure 7A, 7B). Compared to *WT* cells, *GCN5* deletion led to ~2-fold decrease of H3K9ac in glucose-replete (EXP) condition, ~3.5-fold reduction upon glucose depletion (PDS) and remarkably, ~14-fold decline during the transition into stationary phase (Figure 7A, 7B). These data further support that Gcn5 plays a feed-forward role in promoting acetyl-CoA synthesis and hence H3K9 acetylation. Previously, the HDA1 complex was reported to deacetylate K9, K14, K18, K23, and K27 on histone H3 that are acetylated by Gcn5 [57]. Indeed, global H3K9ac levels were increased in the *hda1Δ* mutant cells by ~40% in the glucose-replete (EXP) conditions and more drastically by ~100% upon glucose depletion (PDS) and during the transition into stationary phase (Figure 7A, 7B). *HDA1* removal from the *gcn5Δ* mutants, however, only marginally enhanced the levels of H3K9ac at the glucose-replete conditions and had no impact on H3K9ac levels in glucose-depleted *gcn5Δ* mutants (Figure 7A, 7B). In contrast to H3K9ac, H3K14ac levels were modestly reduced in the *gcn5Δ* or *gcn5Δhda1Δ* mutants, and not significantly enhanced in the *hda1Δ* cells across the three growth phases (Figure 7C). These data support that the HDA1 complex regulates H3K9 acetylation in a manner dependent on Gcn5.

Global H3K9ac levels in transition-phase cells were found to inversely correlate with the percentage of viable cells capable of quiescence exit at the late-stationary phase (Figure 7D) when the majority of the ageing cells remained viable (Figure 3B), suggesting that decreased H3K9 acetylation in *gcn5Δ* may prevent chronologically ageing cells from entering into senescence, thus retaining their CFU ability (Figure 3C). Conversely, Hda1 removal led to enhanced acetylation, accompanied by a significant decrease of clonogenic survival among the viable populations towards the late-stationary phase (Figures 3C, 7D). Put together, these data suggest that the readouts of Gcn5-mediated histone acetylation (stress resistance and redox homeostasis) and histone acetylation status itself have opposing roles in CLS regulation. These two characteristics together ultimately determine the dynamics of lifespan in non-dividing cells of yeast (Figure 3A).

## DISCUSSION

**Figure 8:**
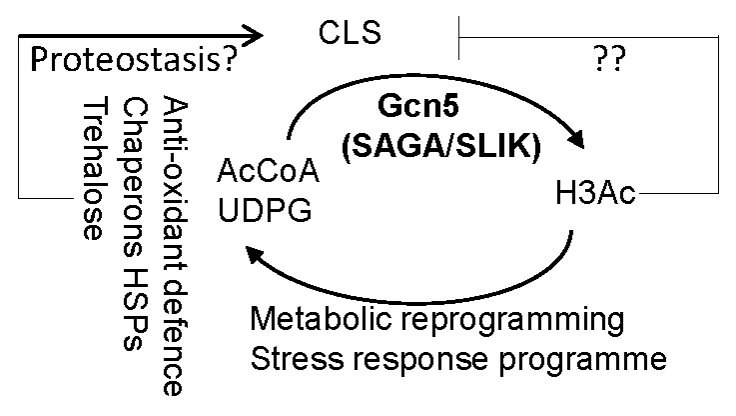
**The working model demonstrating the contradictory roles of Gcn5 in CLS regulation.** AcCoA: acetyl-CoA. UDPG: UDP-glucose ? and ?? denote respectively the anti-ageing and pro-ageing hallmarks to be determined. Arrow: activation; bar: inhibition.

Ageing is inevitable but also malleable. The antagonistic pleiotropy theory of ageing [58, 59], posits that the process of ageing is predominantly determined by the action of wild-type genes that favour growth and development in early life. Recently, epigenetic alterations, which are set in motion during development or in response to cellular damage, have been proposed as the major diver of ageing [60–62]. Here, we report that Gcn5 in SAGA/SLIK has such antagonistic pleiotropic effects on chronological ageing in yeast (Figure 8). On the one hand, Gcn5 is not only necessary for the basal levels of H3K9 acetylation and fermentative growth but also essential to maintaining and/or elevating H3K9ac levels at the target genes in glucose-depleted cells (Figure 6) or in cells under acute glucose withdrawal [54], activating the stress response program and metabolic reprogramming (Figure 4), cellular stress resistance and redox homeostasis (Figure 5) and as a result, CLS extension of early-/mid-stationary phase cells (Figure 3). On the other, Gcn5- mediated metabolic reprogramming also includes the PDH bypass (Figure 4), leading to acetyl-CoA synthesis (Figure 6F) and global histone acetylation status which is positively correlated with senescence entry (Figure 7). These data suggest that there are two counteracting factors regulating senescence entry/CFU potential: the capability of stress resistance and the acetylation states of the chromatin (Figure 8). Trehalose and glycogen acquired during the transition to stationary phase are mobilised to support the anti-oxidant defence system and quiescence exit [63–65]. Similarly, the molecular chaperones expressed during the transition phases have limited half-lives. Thus, the sharp reduction of CFU potential observed in WT cell cultures after day 24 suggests that WT cells may have gradually exhausted their stress resistance capability before late-stationary phase (Figure 3A, 3C) yet retaining their acetylation status, prompting rapid entry into the senescent state thereafter. Such hypothesis needs to be experimentally confirmed but is supported by the observation that the *hda1Δ* mutants (similar stress resistance capability but higher acetylation levels than WT cells, Figures 5, 7) had similar CFU potential in the first 24 days but lost their CFU potential more quickly than WT cells (Figure 3A, 3C). The *gcn5Δ* or *gcn5Δhda1Δ* mutants (with compromised stress resistance but lower acetylation levels, Figures 5, 7) lost their CFU potential early but at a much slower rate than the late-stationary phase WT or *hda1Δ* cells (Figure 3A, 3C).

Gcn5-mediated anti-ageing characteristics include the expression of molecular chaperones and hydrophillins (Table 1 and Figure 1), the activation of anti-oxidant defence system and redox homeostasis, and to a lesser extent, the accumulation of trehalose (Figure 5), the latter of which has been shown to be positively correlated with proteostasis and/or CLS extension in previous studies [2, 21, 63, 66, 67]. Molecular chaperones, mostly represented by HSPs that mediate heat shock response (HSR), are able to recognize and disperse stress-triggered biomolecular condensates directly and efficiently in yeast [68, 69]. The expression of HSPs and coordination of HSR are required for lifespan extension in *C. elegans* in response to reduced insulin/IGF-1-like signalling or dietary restriction [70–72]. Thus, the readouts of Gcn5-mediated acetylation may function to maintain cellular proteostasis to enable lifespan extension of stationary phase cells (Figures 3A, 8).

Emerging evidence has suggested epigenetic regulation, including histone modifications, acts as a driving force of cellular senescence [73–75]. How histone acetylation might promote senescence entry of chronologically ageing cells (Figure 8), however, is currently unknown. Histone acetylation facilitates open chromatin and active gene transcription [76, 77]. Histone H3K9 acetylation is characteristic of a more relaxed chromatin state [78]. Conversely, hypoacetylated H3K9, H3K14 and H4K16 are associated with silenced chromatin, recombination suppression, and a delay in entry into replicative senescence [79]. Inhibiting histone acetyltransferase activity of Gcn5 extends RLS in both yeast and human cell lines in a manner dependent on reduced H3K9 and H3K18 acetylation [13]. These data suggest that hypoacetylation in the *gcn5Δ* mutants may delay senescence entry through transcription silencing and hence enhanced genomic stability. Furthermore, spermidine treatment that inhibits HAT activities leads to H3 hypoacetylation, autophagy induction and CLS extension [80]. Depleting acetyl-CoA levels by knocking down the growth-essential acetyl-CoA synthetase *ACS2* compromises H3 acetylation at K9, K14 and K18, leading to increased autophagy and CLS extension in yeast [81]. Thus, hypoacetylation in the *gcn5Δ* mutants may act to extend CLS by enhancing autophagy, counteracting the proteostasis defects due to loss of redox homeostasis and compromised stress resistance (Figures 1, 5), and therefore a slower rate of CLS decline (Figure 3A). Future work is necessary to address the mechanisms underlying CLS extension in hypoacetylated cells. Nevertheless, a recent report has revealed that in *Caenorhabditis elegans*, mitochondrial stress-induced longevity is mediated through nuclear accumulation of NuRD histone deacetylase and chromatin remodelling in response to reduced acetyl-CoA levels [82], indicating that reducing acetyl-CoA synthesis and histone acetylation also retards organismal ageing. Given that mammalian Gcn5 is essential for developmental processes [83, 84], modulating Gcn5 acetyltransferase activity and/or histone H3K9ac levels could be an effective means to delay senescence entry and to improve lifespan at a later stage in life. Interestingly, Gcn5 has also been shown to be overexpressed in numerous types of cancer cells (colon cancer, Burkitt lymphoma, and lung cancer), in which it functions as a transcription co-activator of the *MYC* oncogene to promote cell growth [85]. Thus, inhibiting the Gcn5/SAGA activity may also be a potential therapeutic strategy to combat cancer.

## MATERIALS AND METHODS

### Strains and plasmids

Strains carrying single-gene deletions were obtained directly from the BY4741/BY4742 mutant libraries (Open Biosystems). Strains carrying deletions of *GCN5* and/or *HDA1* were regenerated by PCR-mediated gene replacement using drug resistant markers. Fluorescent reporter plasmids, pHSP26-HSP26-RFP/VFP and pSSA3-RFP/VFP, were constructed previously [21]. The FBP1-GFP expression plasmid was constructed by cloning FBP1-GFP coding sequences between the *FBP1* promoter and the *ADH1* terminator in pRS425.

### Fluorescent reporter assays

Cells bearing reporter constructs were grown in appropriate synthetic dropout medium overnight, followed by inoculation into 5ml fresh dropout medium (0.6% glucose) in 50ml falcon tubes to achieve a starting OD_600nm_ of 0.2. At 6-, 12-, 24-, and 48-hour post-inoculation, around 1x10^7 cells were harvested by centrifugation and resuspended in 1ml of 1x PBS (pH7.4) for fluorescence quantification using BD LSRFortessaTM (BD Biosciences, USA) or Attune Nxt (Thermo Fisher Scientific, USA) instruments. RFP was measured using a 561nm excitation laser and a 610/20nm (BD LSRFortessaTM) or 620/15nm (Attune Nxt) emission filter. VFP was measured using a 488nm excitation laser and a 530/30nm emission filter. The mean fluorescence level was calculated from readings of at least 10,000 cells using the FlowJo software (version 10.2).

### Determination of storage carbohydrates, ROS and stress resistance

Intracellular trehalose and glycogen, ROS levels and spotting assays to measure stress resistance displayed by the early-stationary phase cells (72 hours incubation in YPD medium) were determined as described previously [21]. Cell growth was measured either by the above described spotting assay on YPD (2% glucose) or YPGE (3% glycerol and 1% ethanol) or using a plate reader (BMG Labtech, USA).

### Chronological lifespan assays

CLS was determined by clonogenicity to access the ability of stationary phase cells to exit from quiescence to form colonies (colony forming units, CFU), and by cell viability to measure the population of cells remaining alive in the stationary phase cultures. Yeast cells were inoculated in YPD medium and incubated for 3 days to early-stationary phase (CLS day 0). Clonogenicity was measured at day 0 and thereafter every 3 or 6 days. The number of CFU was scored by spreading cells onto YPD plates in triplicates after serial dilutions (1:20, 1:50, and 1:100). Relative CFU was calculated by normalising the mean CFU to that at day 0. Cell viability was determined by flow cytometry analysis of SYTOX Green-stained cells, as described previously [21].

### Protein isolation and Western blotting

Cells grown to exponential (6h), early post-diauxic shift (14h) and during the transition to stationary phase (24h) in YPD medium were harvested by centrifugation, washed once with ice-cold lysis buffer (20mM HEPES, pH 7.5, 300mM NaCl, 10% (v/v) glycerol), snap-frozen with liquid nitrogen and stored at -80° C until cell lysis. Cells were lysed in lysis buffer containing 1x cOmplete™ proteinase inhibitor cocktail (Roche, Switzerland) by beads beating with acid-washed glass beads (425~600μm, Sigma-Aldrich, USA) using a FastPrep-24™ 5G instrument (MB Biochemical). Following 10 minutes of centrifugation at 4° C to remove pellet, total protein concentration was measured with Pierce™ BCA Protein Assay Kit (Thermo Fisher Scientific) and diluted to a concentration of 2mg/ml with lysis buffer. Proteins were denatured by incubating at 95° C for 10 minutes with 2x Laemmli buffer, and 20μg was applied to a 15% Tris-glycine gel for separation. The transfer process was performed using the Trans-Blot Turbo transfer system (Bio-Rad Laboratories, USA) with a 0.2μm PVDF transfer pack (Bio-Rad Laboratories). Membranes were incubated with 6% (w/v) BSA in TBST solution (0.1% Tween 20) for 2 hours at room temperature, followed by primary antibody incubation overnight at 4° C and subsequent secondary antibody incubation for 1 hour at room temperature. The membrane was scanned using an Odyssey® CLx imaging system (Li-COR, USA) and densitometry analysis of protein bands was performed using the Image Studio software (Version 5.2.5, Li-COR). Primary antibodies used were Anti-H3K9ac (1:5,000, 07-352, Upstate®), Anti-H3K14ac (1:5,000, 07-353, Upstate®) and Anti-H3 (1:5,000, ab1791, Abcam). Secondary antibodies used were IRDye® 680RD Goat anti-Rabbit IgG Secondary Antibody (1:20,000, 925-68071, Li-COR).

### Chromatin immunoprecipitation (ChIP)

Cells grown to exponential and early post-diauxic shift phases in YPD medium were crosslinked with 1% formaldehyde at 30° C for 20 minutes. Crosslinking reaction was stopped by adding 2.5M glycine to a final concentration of 125mM and incubated for 5 minutes. Cells were harvested by centrifugation and washed once with FA lysis buffer (50mM HEPES-KOH, pH 7.5, 150Mm NaCl, 1mM EDTA, pH 7.6, 1% (v/v) Triton X-100, 0.1% (w/v) sodium deoxycholate and 0.1% (w/v) SDS), snap-frozen and stored at -80° C. Cell lysis was performed in cold FA lysis buffer containing cOmplete™ proteinase inhibitor cocktail (Roche) by beads beating with acid-washed glass beads (425~600μm, Sigma-Aldrich) using a FastPrep-24™ 5G instrument (MB Biochemical). Chromatin was sheared by sonication on a Bioruptor Plus (Diagnode) at the high power setting for 24 cycles (30sec ON/OFF). After centrifugation for 10 minutes at 10,000x g, 4° C, supernatants were transferred to fresh tubes. A similar amount of chromatin equivalent to 150μg of total protein was used for immunoprecipitation (IP) with 3μg of anti-H3K9ac (07-352, Upstate®) or anti-H3 (ab1791, Abcam) overnight at 4° C on a rotation wheel. 40ul of pre-equilibrated Protein A/G Sepharose® (ab193262, Abcam) beads were added to each IP sample and incubated for 5 hours at 4° C. Subsequently, beads were washed thrice with FA lysis buffer, twice with wash buffer 1 (FA lysis buffer containing 0.5M NaCl), and twice with wash buffer 2 (10mM Tris-HCl, pH 8.0, 0.25M LiCl, 1mM EDTA, 0.5% (v/v) NP-40, 0.5% (w/v) sodium deoxycholate), then eluted overnight at 65° C with 130μl of 1% SDS-TE buffer (100mM Tris-HCl, pH 8.0, 10mM EDTA, 1% (v/v) SDS) with 5μg/ml RNase A (Roche). Simultaneously, 30μl of 1% SDS-TE buffer with 5μg/ml RNase A (Roche) was added to the input sample and incubated overnight at 65° C. IP and input samples were treated with 100μg of proteinase K (Roche) for 1 hour at 55° C, then purified with the QIAquick PCR purification kit (Qiagen). Purified DNAs were used as template to quantify the targets bound by anti-H3K9ac or anti-H3 using the QuantiFast SYBR® Green PCR Kit (Qiagen) on Rotor-Gene 6000 (Corbett Research, UK). ChIP signals were normalised to input to generate percent input for subsequent analysis.

### RNA-seq analysis

Total RNA was isolated from exponentially growing and early post-diauxic phase cells using a RNeasy Mini kit (Qiagen). PolyA-enriched RNA samples were sequenced on Illumina Hiseq platform by Novogen UK limited. The raw transcriptome data are available at Biostudies (https://www.ebi.ac.uk/biostudies/arrayexpress) under the accession number: E-MTAB-11046. After quality check with FastQC (version 0.11.5), RNAseq data were mapped to the *S. cerevisiae* reference genome R64-1-1 using HISAT2 (version 2.1.0) [86]. Quality of reads alignment was further checked with Picard Tools (version 1.96) and MultiQC (version 1.8), followed by quantification of reads overlapping coding genes by the featureCounts programme [87]. DESeq2 (version 1.24.0) analyses [88] were performed within R (version 3.6.1), comparing the changes in gene expression. Hierarchical clustering was performed using the ComplexHeatmap package (version 2.4.2) [89]. Visualisation of expression of genes within a KEGG pathway was generated by the Pathview package (version 1.28.0) [90].

### Metabolites extraction and targeted metabolite analysis (LC-MS/MS)

Intracellular metabolites were extracted following a protocol adapted from previous publications [91, 92]. Cells were grown in YPD medium (2% glucose) until early-post diauxic shift phase. A culture containing cells equivalent to 3 units of OD_600nm_ was aliquoted and injected into a 15ml tube containing 5 volumes of pure methanol precooled to -40° C (final methanol concentration of 83%) for quenching, followed by centrifugation to pellet cells. At the same time, a replicate cell sample was harvested for protein quantification. A 60 μL of an internal standard mix at 10 μM (AMP _13_C^10^, _15_N^5^; ATP _13_C^10^, _15_N^5^; Glutamate U^13^C, U^15^N; Leucine-d_10_, Phenylalanine-d_5_, Proline U^13^C, U^15^N; and Valine-d_8_), was added to cell pellets, followed by resuspension in 2ml of -20° C extraction buffer (methanol:acetonitrile:water, 40:40:20). Metabolites were extracted from cells via sonication in a 4° C water bath for 30 minutes and four freeze-thaw cycles between -20° C and -80° C. The mixture was transferred into a new 2ml screw-cap tube for centrifugation at 16,100x g for 15 minutes at 4° C to pellet the cells. The supernatant was aliquoted into a fresh 2ml screw-cap tube to dry under a flow of nitrogen and stored at -80° C until analysis.

The dried metabolites were reconstituted in 100μL of 10 mM ammonium carbonate in acetonitrile:water mix (7:3). Samples were injected onto a UHPLC^+^ series coupled to a TSQ Quantiva mass spectrometer (Thermo Fisher Scientific, USA) using a heated ESI source. The electrospray voltage was set to 3.5 kV and 2.5 kV for positive and negative ionisation modes, respectively. Sheath gas was set at 52 (arbitrary units), auxiliary gas at 16, sweep gas at 2, and ion transfer tube at 356° C. Samples were analysed using two different chromatographic methods, normal phase, and reverse-phase analysis. For normal phase analysis*,* the samples were analysed with a BEH-amide HILIC (150 x 2.1 mm 1.7 μm) column at 30° C. The mobile phase consisted of: (A) 0.1% of ammonium carbonate and (B) acetonitrile and was pumped at a flow rate of 0.6 mL/min. The gradient was programmed as follows: 80 % of B for 1.50 min followed by a linear decrease from 80 % to 40 % of B for 3.5 min and finally returned to initial conditions. For reverse phase analysis*:* Samples were dried and reconstituted in 10 mM ammonium acetate solution and analysed with an ACE C18 PFP (150 x 2.1 mm 5 μm) column at 30° C. The mobile phase consisted of (A) 0.1 % formic acid in water and (B) 0.1 % formic acid in acetonitrile, pumped at 0.5 mL/min. The gradient was programmed as follows: 0 % of B for 1.60 min followed by a linear increase from 0 % to 30 % of B for 4 min and 90 % by 4.5 min, held for 1 min and then returned to initial conditions.

Calculated masses and mass fragments of the calculated compounds are reported in Supplementary Table 2. Data were processed using Xcalibur software (Thermo Fisher Scientific), and peak intensities of target metabolites were normalised to an appropriate internal standard and then to protein content, which was quantified following the same protocol as described for western blotting.

## Supplementary Material

Supplementary Figures

Supplementary Table 1

Supplementary Table 2

## References

[1] Fabrizio P, Longo VD. The chronological life span of Saccharomyces cerevisiae. Aging Cell. 2003; 2:73–81. 10.1046/j.1474-9728.2003.00033.x 12882320

[2] Mirisola MG, Longo VD. Yeast Chronological Lifespan: Longevity Regulatory Genes and Mechanisms. Cells. 2022; 11:1714. 10.3390/cells11101714 35626750 PMC9139625

[3] Capuano F, Mülleder M, Kok R, Blom HJ, Ralser M. Cytosine DNA methylation is found in Drosophila melanogaster but absent in Saccharomyces cerevisiae, Schizosaccharomyces pombe, and other yeast species. Anal Chem. 2014; 86:3697–702. 10.1021/ac500447w 24640988 PMC4006885

[4] Molina-Serrano D, Kyriakou D, Kirmizis A. Histone Modifications as an Intersection Between Diet and Longevity. Front Genet. 2019; 10:192. 10.3389/fgene.2019.00192 30915107 PMC6422915

[5] Yi SJ, Kim K. New Insights into the Role of Histone Changes in Aging. Int J Mol Sci. 2020; 21:8241. 10.3390/ijms21218241 33153221 PMC7662996

[6] Sen P, Dang W, Donahue G, Dai J, Dorsey J, Cao X, Liu W, Cao K, Perry R, Lee JY, Wasko BM, Carr DT, He C, et al. H3K36 methylation promotes longevity by enhancing transcriptional fidelity. Genes Dev. 2015; 29:1362–76. 10.1101/gad.263707.115 26159996 PMC4511212

[7] Schroeder EA, Raimundo N, Shadel GS. Epigenetic silencing mediates mitochondria stress-induced longevity. Cell Metab. 2013; 17:954–64. 10.1016/j.cmet.2013.04.003 23747251 PMC3694503

[8] Lim S, Ahn H, Duan R, Liu Y, Ryu HY, Ahn SH. The Spt7 subunit of the SAGA complex is required for the regulation of lifespan in both dividing and nondividing yeast cells. Mech Ageing Dev. 2021; 196:111480. 10.1016/j.mad.2021.111480 33831401

[9] Denoth-Lippuner A, Krzyzanowski MK, Stober C, Barral Y. Role of SAGA in the asymmetric segregation of DNA circles during yeast ageing. Elife. 2014; 3:e03790. 10.7554/eLife.03790 25402830 PMC4232608

[10] Kim S, Ohkuni K, Couplan E, Jazwinski SM. The histone acetyltransferase GCN5 modulates the retrograde response and genome stability determining yeast longevity. Biogerontology. 2004; 5:305–16. 10.1007/s10522-004-2568-x 15547318

[11] Jiang JC, Stumpferl SW, Tiwari A, Qin Q, Rodriguez-Quiñones JF, Jazwinski SM. Identification of the Target of the Retrograde Response that Mediates Replicative Lifespan Extension in Saccharomyces cerevisiae. Genetics. 2016; 204:659–73. 10.1534/genetics.116.188086 27474729 PMC5068853

[12] McCormick MA, Mason AG, Guyenet SJ, Dang W, Garza RM, Ting MK, Moller RM, Berger SL, Kaeberlein M, Pillus L, La Spada AR, Kennedy BK. The SAGA histone deubiquitinase module controls yeast replicative lifespan via Sir2 interaction. Cell Rep. 2014; 8:477–86. 10.1016/j.celrep.2014.06.037 25043177 PMC4284099

[13] Huang B, Zhong D, Zhu J, An Y, Gao M, Zhu S, Dang W, Wang X, Yang B, Xie Z. Inhibition of histone acetyltransferase GCN5 extends lifespan in both yeast and human cell lines. Aging Cell. 2020; 19:e13129. 10.1111/acel.13129 32157780 PMC7189995

[14] Picazo C, Orozco H, Matallana E, Aranda A. Interplay among Gcn5, Sch9 and mitochondria during chronological aging of wine yeast is dependent on growth conditions. PLoS One. 2015; 10:e0117267. 10.1371/journal.pone.0117267 25658705 PMC4319768

[15] Herman PK. Stationary phase in yeast. Curr Opin Microbiol. 2002; 5:602–7. 10.1016/s1369-5274(02)00377-6 12457705

[16] Gray JV, Petsko GA, Johnston GC, Ringe D, Singer RA, Werner-Washburne M. “Sleeping beauty”: quiescence in Saccharomyces cerevisiae. Microbiol Mol Biol Rev. 2004; 68:187–206. 10.1128/MMBR.68.2.187-206.2004 15187181 PMC419917

[17] De Virgilio C. The essence of yeast quiescence. FEMS Microbiol Rev. 2012; 36:306–39. 10.1111/j.1574-6976.2011.00287.x 21658086

[18] Zhang N, Cao L. Starvation signals in yeast are integrated to coordinate metabolic reprogramming and stress response to ensure longevity. Curr Genet. 2017; 63:839–43. 10.1007/s00294-017-0697-4 28444510 PMC5605593

[19] Pedruzzi I, Dubouloz F, Cameroni E, Wanke V, Roosen J, Winderickx J, De Virgilio C. TOR and PKA signaling pathways converge on the protein kinase Rim15 to control entry into G0. Mol Cell. 2003; 12:1607–13. 10.1016/s1097-2765(03)00485-4 14690612

[20] Martin DE, Soulard A, Hall MN. TOR regulates ribosomal protein gene expression via PKA and the Forkhead transcription factor FHL1. Cell. 2004; 119:969–79. 10.1016/j.cell.2004.11.047 15620355

[21] Cao L, Tang Y, Quan Z, Zhang Z, Oliver SG, Zhang N. Chronological Lifespan in Yeast Is Dependent on the Accumulation of Storage Carbohydrates Mediated by Yak1, Mck1 and Rim15 Kinases. PLoS Genet. 2016; 12:e1006458. 10.1371/journal.pgen.1006458 27923067 PMC5140051

[22] Quan Z, Cao L, Tang Y, Yan Y, Oliver SG, Zhang N. The Yeast GSK-3 Homologue Mck1 Is a Key Controller of Quiescence Entry and Chronological Lifespan. PLoS Genet. 2015; 11:e1005282. 10.1371/journal.pgen.1005282 26103122 PMC4477894

[23] Wu J, Carmen AA, Kobayashi R, Suka N, Grunstein M. HDA2 and HDA3 are related proteins that interact with and are essential for the activity of the yeast histone deacetylase HDA1. Proc Natl Acad Sci USA. 2001; 98:4391–6. 10.1073/pnas.081560698 11287668 PMC31845

[24] Canzonetta C, Leo M, Guarino SR, Montanari A, Francisci S, Filetici P. SAGA complex and Gcn5 are necessary for respiration in budding yeast. Biochim Biophys Acta. 2016; 1863:3160–8. 10.1016/j.bbamcr.2016.10.002 27741413

[25] Warfield L, Ranish JA, Hahn S. Positive and negative functions of the SAGA complex mediated through interaction of Spt8 with TBP and the N-terminal domain of TFIIA. Genes Dev. 2004; 18:1022–34. 10.1101/gad.1192204 15132995 PMC406292

[26] Belotserkovskaya R, Sterner DE, Deng M, Sayre MH, Lieberman PM, Berger SL. Inhibition of TATA-binding protein function by SAGA subunits Spt3 and Spt8 at Gcn4-activated promoters. Mol Cell Biol. 2000; 20:634–47. 10.1128/MCB.20.2.634-647.2000 10611242 PMC85153

[27] Pray-Grant MG, Schieltz D, McMahon SJ, Wood JM, Kennedy EL, Cook RG, Workman JL, Yates JR 3rd, Grant PA. The novel SLIK histone acetyltransferase complex functions in the yeast retrograde response pathway. Mol Cell Biol. 2002; 22:8774–86. 10.1128/MCB.22.24.8774-8786.2002 12446794 PMC139885

[28] Spedale G, Mischerikow N, Heck AJR, Timmers HTM, Pijnappel WW. Identification of Pep4p as the protease responsible for formation of the SAGA-related SLIK protein complex. J Biol Chem. 2010; 285:22793–9. 10.1074/jbc.M110.108787 20498363 PMC2906270

[29] Espinola-Lopez JM, Tan S. The Ada2/Ada3/Gcn5/Sgf29 histone acetyltransferase module. Biochim Biophys Acta Gene Regul Mech. 2021; 1864:194629. 10.1016/j.bbagrm.2020.194629 32890768 PMC8351874

[30] Boyer LA, Langer MR, Crowley KA, Tan S, Denu JM, Peterson CL. Essential role for the SANT domain in the functioning of multiple chromatin remodeling enzymes. Mol Cell. 2002; 10:935–42. 10.1016/s1097-2765(02)00634-2 12419236

[31] Ocampo A, Barrientos A. Quick and reliable assessment of chronological life span in yeast cell populations by flow cytometry. Mech Ageing Dev. 2011; 132:315–23. 10.1016/j.mad.2011.06.007 21736893

[32] Yu R, Cao X, Sun L, Zhu JY, Wasko BM, Liu W, Crutcher E, Liu H, Jo MC, Qin L, Kaeberlein M, Han Z, Dang W. Inactivating histone deacetylase HDA promotes longevity by mobilizing trehalose metabolism. Nat Commun. 2021; 12:1981. 10.1038/s41467-021-22257-2 33790287 PMC8012573

[33] Thomas-Chollier M, Sand O, Turatsinze JV, Janky R, Defrance M, Vervisch E, Brohée S, van Helden J. RSAT: regulatory sequence analysis tools. Nucleic Acids Res. 2008; 36:W119–27. 10.1093/nar/gkn304 18495751 PMC2447775

[34] Monteiro PT, Oliveira J, Pais P, Antunes M, Palma M, Cavalheiro M, Galocha M, Godinho CP, Martins LC, Bourbon N, Mota MN, Ribeiro RA, Viana R, et al. YEASTRACT+: a portal for cross-species comparative genomics of transcription regulation in yeasts. Nucleic Acids Res. 2020; 48:D642–9. 10.1093/nar/gkz859 31586406 PMC6943032

[35] Shore D, Zencir S, Albert B. Transcriptional control of ribosome biogenesis in yeast: links to growth and stress signals. Biochem Soc Trans. 2021; 49:1589–99. 10.1042/BST20201136 34240738 PMC8421047

[36] Cai L, McCormick MA, Kennedy BK, Tu BP. Integration of multiple nutrient cues and regulation of lifespan by ribosomal transcription factor Ifh1. Cell Rep. 2013; 4:1063–71. 10.1016/j.celrep.2013.08.016 24035395 PMC3792855

[37] Downey M, Knight B, Vashisht AA, Seller CA, Wohlschlegel JA, Shore D, Toczyski DP. Gcn5 and sirtuins regulate acetylation of the ribosomal protein transcription factor Ifh1. Curr Biol. 2013; 23:1638–48. 10.1016/j.cub.2013.06.050 23973296 PMC3982851

[38] Deminoff SJ, Santangelo GM. Rap1p requires Gcr1p and Gcr2p homodimers to activate ribosomal protein and glycolytic genes, respectively. Genetics. 2001; 158:133–43. 10.1093/genetics/158.1.133 11333224 PMC1461654

[39] Lieb JD, Liu X, Botstein D, Brown PO. Promoter-specific binding of Rap1 revealed by genome-wide maps of protein-DNA association. Nat Genet. 2001; 28:327–34. 10.1038/ng569 11455386

[40] Zhang N, Oliver SG. The transcription activity of Gis1 is negatively modulated by proteasome-mediated limited proteolysis. J Biol Chem. 2010; 285:6465–76. 10.1074/jbc.M109.073288 20022953 PMC2825442

[41] Amorós M, Estruch F. Hsf1p and Msn2/4p cooperate in the expression of Saccharomyces cerevisiae genes HSP26 and HSP104 in a gene- and stress type-dependent manner. Mol Microbiol. 2001; 39:1523–32. 10.1046/j.1365-2958.2001.02339.x 11260469

[42] Orzechowski Westholm J, Tronnersjö S, Nordberg N, Olsson I, Komorowski J, Ronne H. Gis1 and Rph1 regulate glycerol and acetate metabolism in glucose depleted yeast cells. PLoS One. 2012; 7:e31577. 10.1371/journal.pone.0031577 22363679 PMC3283669

[43] Pedruzzi I, Bürckert N, Egger P, De Virgilio C. Saccharomyces cerevisiae Ras/cAMP pathway controls post-diauxic shift element-dependent transcription through the zinc finger protein Gis1. EMBO J. 2000; 19:2569–79. 10.1093/emboj/19.11.2569 10835355 PMC212766

[44] Zhang N, Quan Z, Rash B, Oliver SG. Synergistic effects of TOR and proteasome pathways on the yeast transcriptome and cell growth. Open Biol. 2013; 3:120137. 10.1098/rsob.120137 23697803 PMC3866871

[45] Zhang N, Wu J, Oliver SG. Gis1 is required for transcriptional reprogramming of carbon metabolism and the stress response during transition into stationary phase in yeast. Microbiology (Reading). 2009; 155:1690–8. 10.1099/mic.0.026377-0 19383711

[46] Tachibana C, Yoo JY, Tagne JB, Kacherovsky N, Lee TI, Young ET. Combined global localization analysis and transcriptome data identify genes that are directly coregulated by Adr1 and Cat8. Mol Cell Biol. 2005; 25:2138–46. 10.1128/MCB.25.6.2138-2146.2005 15743812 PMC1061606

[47] Charbon G, Breunig KD, Wattiez R, Vandenhaute J, Noël-Georis I. Key role of Ser562/661 in Snf1-dependent regulation of Cat8p in Saccharomyces cerevisiae and Kluyveromyces lactis. Mol Cell Biol. 2004; 24:4083–91. 10.1128/MCB.24.10.4083-4091.2004 15121831 PMC400452

[48] Haurie V, Perrot M, Mini T, Jenö P, Sagliocco F, Boucherie H. The transcriptional activator Cat8p provides a major contribution to the reprogramming of carbon metabolism during the diauxic shift in Saccharomyces cerevisiae. J Biol Chem. 2001; 276:76–85. 10.1074/jbc.M008752200 11024040

[49] Abate G, Bastonini E, Braun KA, Verdone L, Young ET, Caserta M. Snf1/AMPK regulates Gcn5 occupancy, H3 acetylation and chromatin remodelling at S. cerevisiae ADY2 promoter. Biochim Biophys Acta. 2012; 1819:419–27. 10.1016/j.bbagrm.2012.01.009 22306658 PMC3319277

[50] Lo WS, Duggan L, Emre NC, Belotserkovskya R, Lane WS, Shiekhattar R, Berger SL. Snf1--a histone kinase that works in concert with the histone acetyltransferase Gcn5 to regulate transcription. Science. 2001; 293:1142–6. 10.1126/science.1062322 11498592

[51] Lo WS, Gamache ER, Henry KW, Yang D, Pillus L, Berger SL. Histone H3 phosphorylation can promote TBP recruitment through distinct promoter-specific mechanisms. EMBO J. 2005; 24:997–1008. 10.1038/sj.emboj.7600577 15719021 PMC554127

[52] Kuo YM, Andrews AJ. Quantitating the specificity and selectivity of Gcn5-mediated acetylation of histone H3. PLoS One. 2013; 8:e54896. 10.1371/journal.pone.0054896 23437046 PMC3578832

[53] Spedale G, Timmers HT, Pijnappel WW. ATAC-king the complexity of SAGA during evolution. Genes Dev. 2012; 26:527–41. 10.1101/gad.184705.111 22426530 PMC3315114

[54] Hsieh WC, Sutter BM, Ruess H, Barnes SD, Malladi VS, Tu BP. Glucose starvation induces a switch in the histone acetylome for activation of gluconeogenic and fat metabolism genes. Mol Cell. 2022; 82:60–74.e5. 10.1016/j.molcel.2021.12.015 34995509 PMC8794035

[55] Krivoruchko A, Zhang Y, Siewers V, Chen Y, Nielsen J. Microbial acetyl-CoA metabolism and metabolic engineering. Metab Eng. 2015; 28:28–42. 10.1016/j.ymben.2014.11.009 25485951

[56] Takahashi H, McCaffery JM, Irizarry RA, Boeke JD. Nucleocytosolic acetyl-coenzyme a synthetase is required for histone acetylation and global transcription. Mol Cell. 2006; 23:207–17. 10.1016/j.molcel.2006.05.040 16857587

[57] Verdone L, Wu J, van Riper K, Kacherovsky N, Vogelauer M, Young ET, Grunstein M, Di Mauro E, Caserta M. Hyperacetylation of chromatin at the ADH2 promoter allows Adr1 to bind in repressed conditions. EMBO J. 2002; 21:1101–11. 10.1093/emboj/21.5.1101 11867538 PMC125900

[58] Williams GC. Pleiotropy, Natural Selection, and the Evolution of Senescence. Evolution. 1957; 11:398–411. 10.2307/2406060

[59] Gems D. The hyperfunction theory: An emerging paradigm for the biology of aging. Ageing Res Rev. 2022; 74:101557. 10.1016/j.arr.2021.101557 34990845 PMC7612201

[60] de Magalhães JP. Ageing as a software design flaw. Genome Biol. 2023; 24:51. 10.1186/s13059-023-02888-y 36973715 PMC10042583

[61] Yang JH, Hayano M, Griffin PT, Amorim JA, Bonkowski MS, Apostolides JK, Salfati EL, Blanchette M, Munding EM, Bhakta M, Chew YC, Guo W, Yang X, et al. Loss of epigenetic information as a cause of mammalian aging. Cell. 2023; 186:305–26.e27. 10.1016/j.cell.2022.12.027 36638792 PMC10166133

[62] Kane AE, Sinclair DA. Epigenetic changes during aging and their reprogramming potential. Crit Rev Biochem Mol Biol. 2019; 54:61–83. 10.1080/10409238.2019.1570075 30822165 PMC6424622

[63] Kyryakov P, Beach A, Richard VR, Burstein MT, Leonov A, Levy S, Titorenko VI. Caloric restriction extends yeast chronological lifespan by altering a pattern of age-related changes in trehalose concentration. Front Physiol. 2012; 3:256. 10.3389/fphys.2012.00256 22783207 PMC3390693

[64] Roy A, Ghosh AK. Correlation between stationary phase survival and acid trehalase activity in yeast. Biochim Biophys Acta. 1998; 1401:235–8. 10.1016/s0167-4889(97)00156-0 9540814

[65] Favre C, Aguilar PS, Carrillo MC. Oxidative stress and chronological aging in glycogen-phosphorylase-deleted yeast. Free Radic Biol Med. 2008; 45:1446–56. 10.1016/j.freeradbiomed.2008.08.021 18804161

[66] Hu J, Wei M, Mirzaei H, Madia F, Mirisola M, Amparo C, Chagoury S, Kennedy B, Longo VD. Tor-Sch9 deficiency activates catabolism of the ketone body-like acetic acid to promote trehalose accumulation and longevity. Aging Cell. 2014; 13:457–67. 10.1111/acel.12202 24649827 PMC4032597

[67] Wei M, Fabrizio P, Hu J, Ge H, Cheng C, Li L, Longo VD. Life span extension by calorie restriction depends on Rim15 and transcription factors downstream of Ras/PKA, Tor, and Sch9. PLoS Genet. 2008; 4:e13. 10.1371/journal.pgen.0040013 18225956 PMC2213705

[68] Yoo H, Bard JAM, Pilipenko EV, Drummond DA. Chaperones directly and efficiently disperse stress-triggered biomolecular condensates. Mol Cell. 2022; 82:741–55.e11. 10.1016/j.molcel.2022.01.005 35148816 PMC8857057

[69] Margulis B, Tsimokha A, Zubova S, Guzhova I. Molecular Chaperones and Proteolytic Machineries Regulate Protein Homeostasis In Aging Cells. Cells. 2020; 9:1308. 10.3390/cells9051308 32456366 PMC7291254

[70] Hsu AL, Murphy CT, Kenyon C. Regulation of aging and age-related disease by DAF-16 and heat-shock factor. Science. 2003; 300:1142–5. 10.1126/science.1083701 12750521

[71] Morley JF, Morimoto RI. Regulation of longevity in Caenorhabditis elegans by heat shock factor and molecular chaperones. Mol Biol Cell. 2004; 15:657–64. 10.1091/mbc.e03-07-0532 14668486 PMC329286

[72] Steinkraus KA, Smith ED, Davis C, Carr D, Pendergrass WR, Sutphin GL, Kennedy BK, Kaeberlein M. Dietary restriction suppresses proteotoxicity and enhances longevity by an hsf-1-dependent mechanism in Caenorhabditis elegans. Aging Cell. 2008; 7:394–404. 10.1111/j.1474-9726.2008.00385.x 18331616 PMC2709959

[73] Pal S, Tyler JK. Epigenetics and aging. Sci Adv. 2016; 2:e1600584. 10.1126/sciadv.1600584 27482540 PMC4966880

[74] Paluvai H, Di Giorgio E, Brancolini C. The Histone Code of Senescence. Cells. 2020; 9:466. 10.3390/cells9020466 32085582 PMC7072776

[75] Crouch J, Shvedova M, Thanapaul RJRS, Botchkarev V, Roh D. Epigenetic Regulation of Cellular Senescence. Cells. 2022; 11:672. 10.3390/cells11040672 35203320 PMC8870565

[76] Barnes CE, English DM, Cowley SM. Acetylation & Co: an expanding repertoire of histone acylations regulates chromatin and transcription. Essays Biochem. 2019; 63:97–107. 10.1042/EBC20180061 30940741 PMC6484784

[77] Grunstein M. Histone acetylation in chromatin structure and transcription. Nature. 1997; 389:349–52. 10.1038/38664 9311776

[78] Nair N, Shoaib M, Sørensen CS. Chromatin Dynamics in Genome Stability: Roles in Suppressing Endogenous DNA Damage and Facilitating DNA Repair. Int J Mol Sci. 2017; 18:1486. 10.3390/ijms18071486 28698521 PMC5535976

[79] Imai S, Armstrong CM, Kaeberlein M, Guarente L. Transcriptional silencing and longevity protein Sir2 is an NAD-dependent histone deacetylase. Nature. 2000; 403:795–800. 10.1038/35001622 10693811

[80] Eisenberg T, Knauer H, Schauer A, Büttner S, Ruckenstuhl C, Carmona-Gutierrez D, Ring J, Schroeder S, Magnes C, Antonacci L, Fussi H, Deszcz L, Hartl R, et al. Induction of autophagy by spermidine promotes longevity. Nat Cell Biol. 2009; 11:1305–14. 10.1038/ncb1975 19801973

[81] Eisenberg T, Schroeder S, Andryushkova A, Pendl T, Küttner V, Bhukel A, Mariño G, Pietrocola F, Harger A, Zimmermann A, Moustafa T, Sprenger A, Jany E, et al. Nucleocytosolic depletion of the energy metabolite acetyl-coenzyme a stimulates autophagy and prolongs lifespan. Cell Metab. 2014; 19:431–44. 10.1016/j.cmet.2014.02.010 24606900 PMC3988959

[82] Zhu D, Wu X, Zhou J, Li X, Huang X, Li J, Wu J, Bian Q, Wang Y, Tian Y. NuRD mediates mitochondrial stress-induced longevity via chromatin remodeling in response to acetyl-CoA level. Sci Adv. 2020; 6:eabb2529. 10.1126/sciadv.abb2529 32789178 PMC7400466

[83] Lin W, Zhang Z, Chen CH, Behringer RR, Dent SY. Proper Gcn5 histone acetyltransferase expression is required for normal anteroposterior patterning of the mouse skeleton. Dev Growth Differ. 2008; 50:321–30. 10.1111/j.1440-169X.2008.01041.x 18430026 PMC4091889

[84] Lin W, Zhang Z, Srajer G, Chen YC, Huang M, Phan HM, Dent SY. Proper expression of the Gcn5 histone acetyltransferase is required for neural tube closure in mouse embryos. Dev Dyn. 2008; 237:928–40. 10.1002/dvdy.21479 18330926 PMC3082922

[85] Koutelou E, Farria AT, Dent SY. Complex functions of Gcn5 and Pcaf in development and disease. Biochim Biophys Acta Gene Regul Mech. 2021; 1864:194609. 10.1016/j.bbagrm.2020.194609 32730897 PMC7854485

[86] Kim D, Paggi JM, Park C, Bennett C, Salzberg SL. Graph-based genome alignment and genotyping with HISAT2 and HISAT-genotype. Nat Biotechnol. 2019; 37:907–15. 10.1038/s41587-019-0201-4 31375807 PMC7605509

[87] Liao Y, Smyth GK, Shi W. featureCounts: an efficient general purpose program for assigning sequence reads to genomic features. Bioinformatics. 2014; 30:923–30. 10.1093/bioinformatics/btt656 24227677

[88] Love MI, Huber W, Anders S. Moderated estimation of fold change and dispersion for RNA-seq data with DESeq2. Genome Biol. 2014; 15:550. 10.1186/s13059-014-0550-8 25516281 PMC4302049

[89] Gu Z, Eils R, Schlesner M. Complex heatmaps reveal patterns and correlations in multidimensional genomic data. Bioinformatics. 2016; 32:2847–9. 10.1093/bioinformatics/btw313 27207943

[90] Luo W, Brouwer C. Pathview: an R/Bioconductor package for pathway-based data integration and visualization. Bioinformatics. 2013; 29:1830–1. 10.1093/bioinformatics/btt285 23740750 PMC3702256

[91] Canelas AB, Ras C, ten Pierick A, van Dam JC, Heijnen JJ, van Gulik WM. Leakage-free rapid quenching technique for yeast metabolomics. Metabolomics. 2008; 4:226–39. 10.1007/s11306-008-0116-4

[92] Rosebrock AP, Caudy AA. Metabolite Extraction from *Saccharomyces cerevisiae* for Liquid Chromatography-Mass Spectrometry. Cold Spring Harb Protoc. 2017; 2017:pdb.prot089086. 10.1101/pdb.prot089086 28864564

