# Supplementary material for: The Saccharomyces cerevisiae acetyltransferase Gcn5 exerts antagonistic pleiotropic effects on chronological ageing
Source: Aging (Albany NY). 2023 Oct 23;15(20):10915–37. doi: 10.18632/aging.205109 (PMC10637828; doi:10.18632/aging.205109)
Supplement: Supplementary Figures [file aging-15-205109-s001.pdf]

SUPPLEMENTARY FIGURES

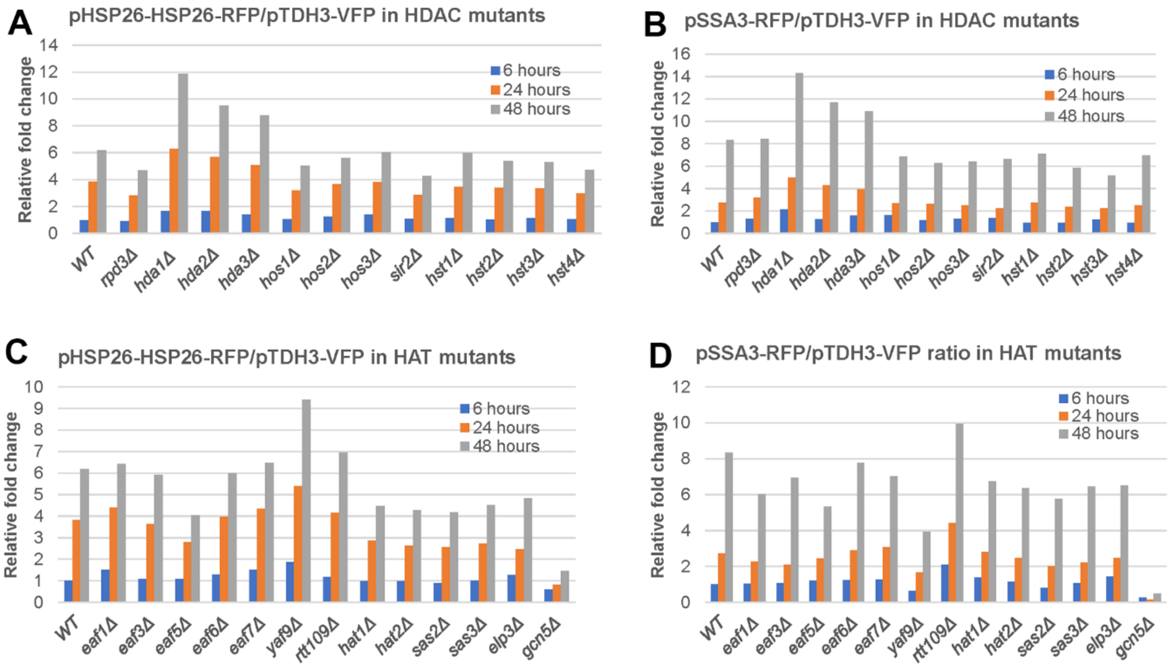

Supplementary Figure 1. Relative pSSA3-RFP and pHSP26-HSP26-RFP levels in HDAC (**A**, **B**) and HAT (**C**, **D**) mutants. The mean RFP fluorescence from 20,000 cells was normalised to that of the pTDH3-VFP at each time point to correct for cell growth differences.

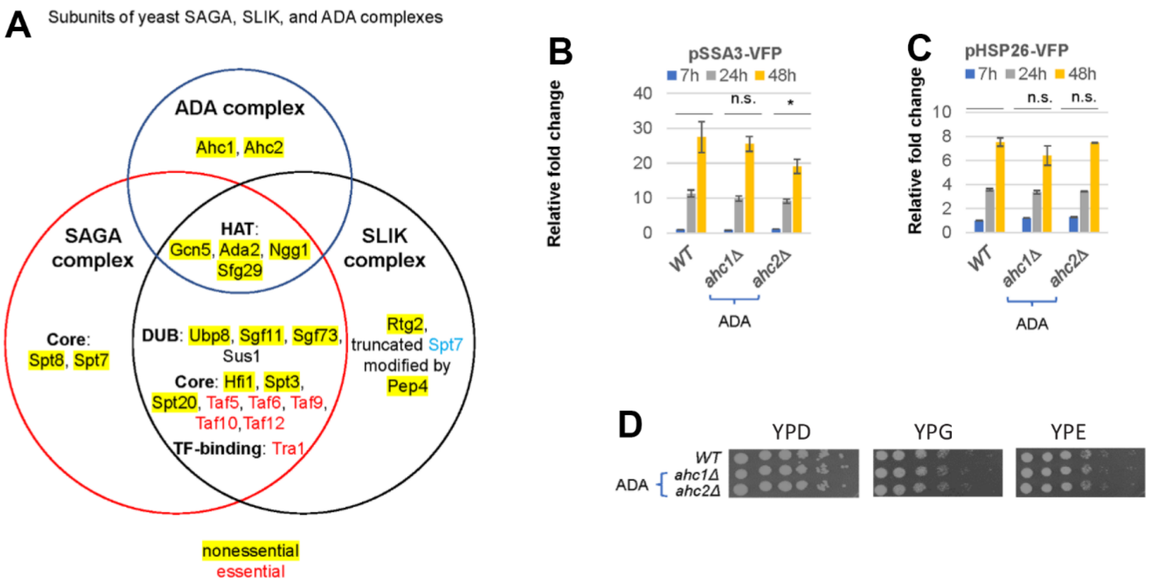

Supplementary Figure 2. (**A**) Schematic illustration of the SAGA/SLIK/ADA complexes; (**B**, **C**) Relative levels of pSSA3-VFP (**B**) and pHSP26-VFP (**C**) in the ADA-specific mutants; (**D**) Spotting assays of cell growth on YPD (2% glucose), YPG (3% glycerol) and YPE (1% Ethanol). Error bars represent standard deviation calculated from biological triplicates. HAT, histone acetyltransferase module; DUB, deubiquitinase module; Core, Core module; SLIK, SAGA-like complex. The significance of difference between *WT* and mutants over all time points was revealed by two-factor ANOVA analysis (***: p < 0.001, **: 0.001<p< 0.01, *: 0.01<p< 0.05 and n.s.: p> 0.05).

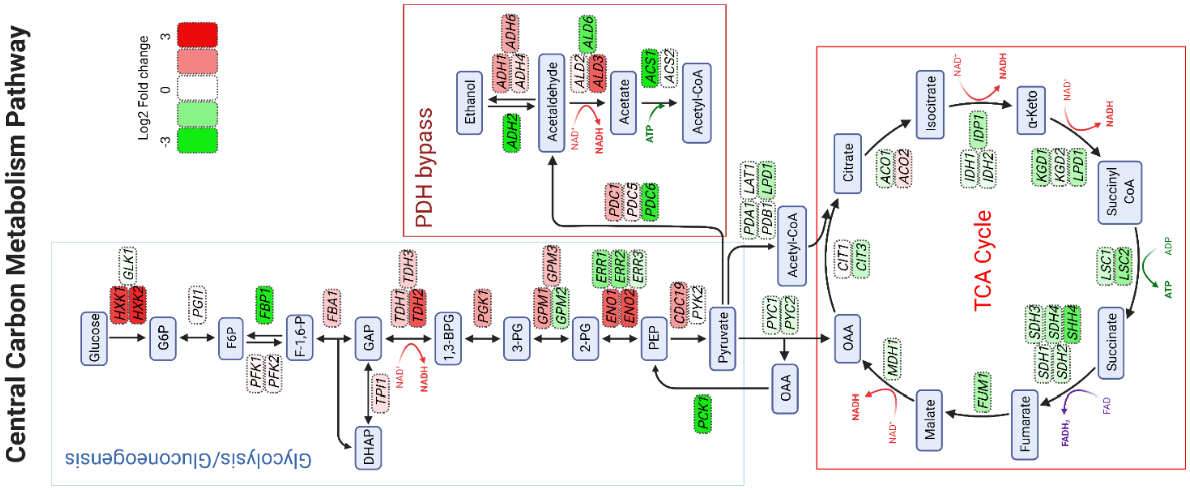

Supplementary Figure 3. KEGG pathway analysis of the differentially expressed genes implicated in central carbon metabolism. Abbreviation: EXP: exponential phase, PDS: early post-diauxic shift phase; G6P: Glucose-6-phosphate, F6P: Fructose-6-phosphate, F-1,6-BP: Fructose-1,6-bisphosphate, F-2,6-BP: Fructose-2,6-bisphosphate, DHAP: Dihydroxy-acetone phosphate, GAP: Glyceraldehyde-3-phosphate, 1,3-BPG: 1,3-bisphosphoglycerate; 3-PG: 3-phosphoglycerate; 2-PG: 2-phosphoglycerate, PEP: Phosphoenolpyruvate, OAA: oxaloacetate. Figure was generated using the BioRender App.

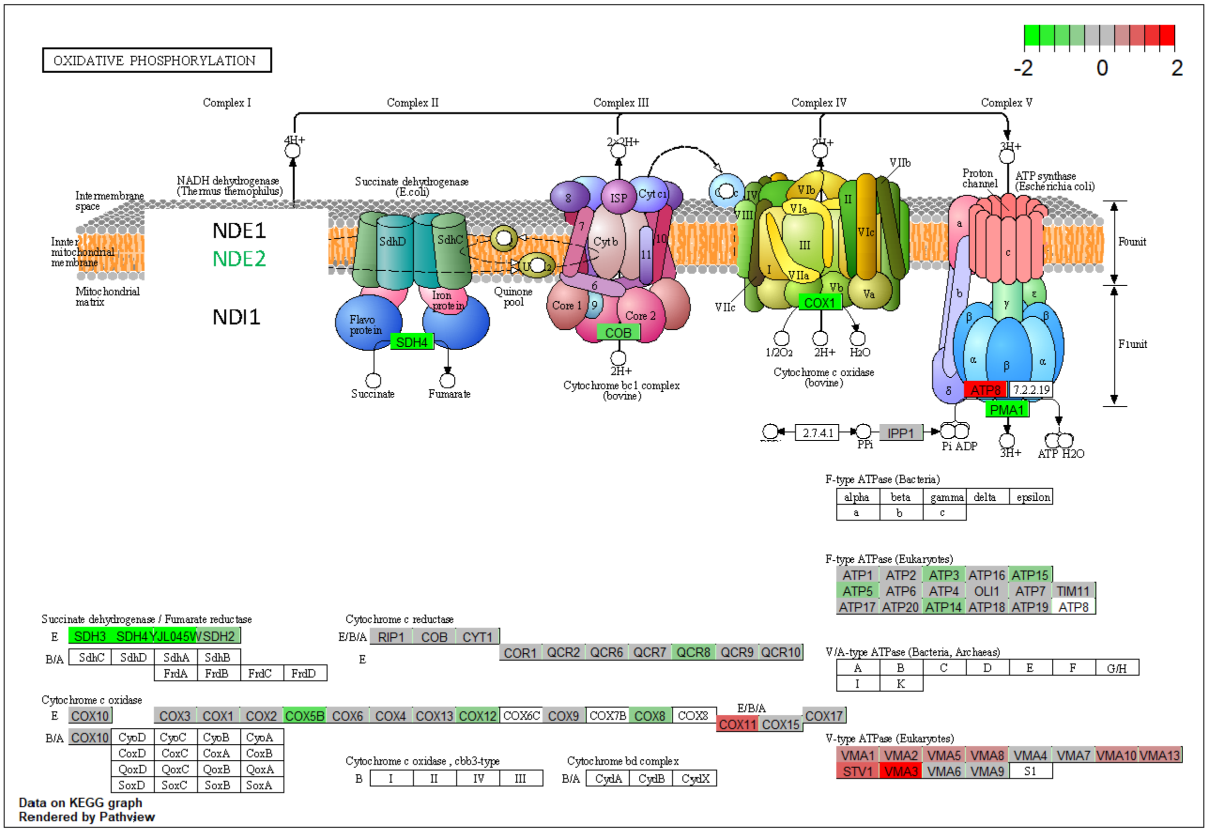

Supplementary Figure 4. Gcn5 promotes the transcription of genes involved in the oxidative phosphorylation in glucose-depleted cells. The oxidative phosphorylation pathway was retrieved from the KEGG website using the pathview package (version 1.28.0) in R. The budding yeast lacks the NADH dehydrogenase complex (complex I) and hence was removed from the image. Instead, budding yeast contains several NADH dehydrogenases, of which the expression of *NDE2* was significantly downregulated in glucose-depleted *gcn5Δ* mutant cells. A colour scale based on log2 fold change indicates changes in the expression levels of other genes.

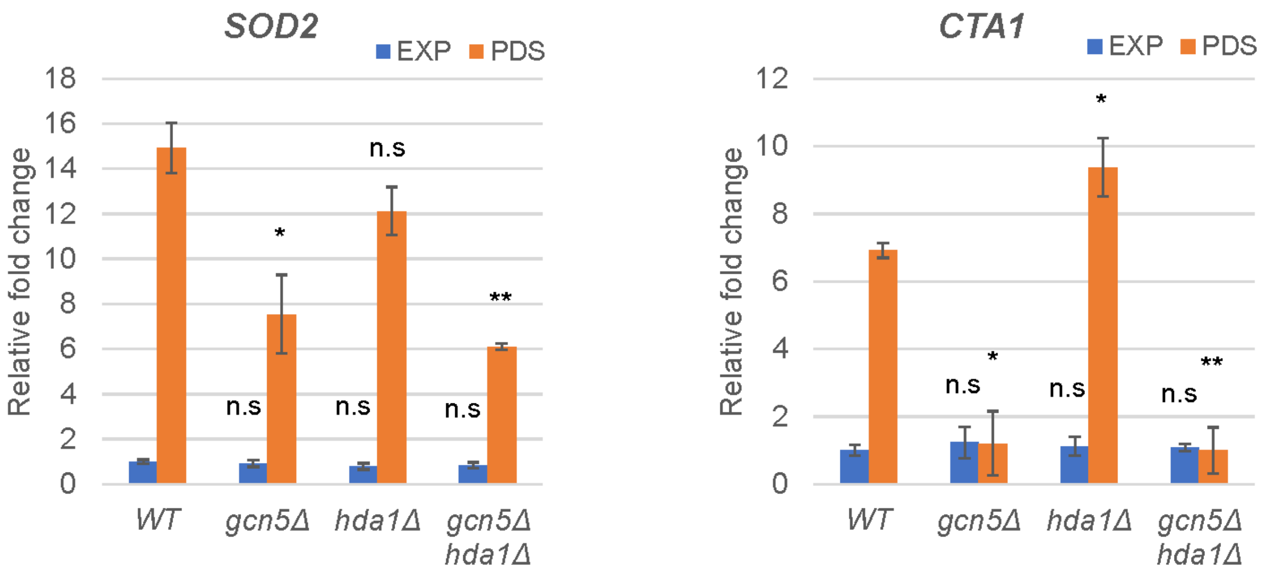

Supplementary Figure 5. Relative levels of *SOD2* and *CTA1* transcripts. The raw read counts of either gene were normalised to that of *ACT1* in the corresponding samples, followed by normalisation to the expression level in *WT* at EXP to calculate relative fold change for comparison among different strains. Error bars represent standard deviation calculated from biological triplicates. Student's t-test was performed to reveal the significance of differences (***: p-value < 0.001, **: p-value < 0.01, *: p-value < 0.05, and n.s.: p-value > 0.05). Abbreviation: EXP, exponential phase; PDS, post-diauxic shift phase.
