# Supplementary material for: The Saccharomyces cerevisiae acetyltransferase Gcn5 exerts antagonistic pleiotropic effects on chronological ageing
Source: Aging (Albany NY). 2023 Oct 23;15(20):10915–37. doi: 10.18632/aging.205109 (PMC10637828; doi:10.18632/aging.205109)
Supplement: Supplementary Table 2 [file aging-15-205109-s003.pdf]

Supplementary Table 2. LC-MS QqQ selected metabolites from the MS/MS analysis.

Metabolite	Column	RT (min)	Mode	Precursor *m/z*	Product *m/z*
**Citrate**	C18PFP	1.32	negative	191.0	111.0
**Fumarate**	C18PFP	1.65	negative	115.0	71.2
**Isocitrate**	C18PFP	1.25	negative	191.0	111.0
**Malate**	C18PFP	1.08	negative	133.0	115.1
**Succinate**	C18PFP	1.82	negative	117.0	73.0
**Acetyl-CoA**	HILIC	3.65	negative	810.0	303.1
**Aconitate**	HILIC	3.47	negative	173.0	85.1
**UDP-Glucose**	HILIC	3.88	negative	565.0	323
**Glucose-6-phosphate**	HILIC	3.66	negative	259.0	97.1
**Phosphoenolpyruvate**	HILIC	3.55	negative	167.0	79.1
**Oxaloacetate**	HILIC	3.34	negative	131.0	87.1
